# Scientific Advances in the Last Decade on the Recovery, Characterization, and Functionality of Bioactive Compounds from the Araticum Fruit (*Annona crassiflora* Mart.)

**DOI:** 10.3390/plants12071536

**Published:** 2023-04-03

**Authors:** Henrique Silvano Arruda, Felipe Tecchio Borsoi, Amanda Cristina Andrade, Glaucia Maria Pastore, Mario Roberto Marostica Junior

**Affiliations:** 1 Bioflavors and Bioactive Compounds Laboratory, Department of Food Science and Nutrition, Faculty of Food Engineering, University of Campinas, Campinas 13083-862, SP, Brazil; felipe.tecchio@gmail.com (F.T.B.); andradenut@gmail.com (A.C.A.);; 2 Nutrition and Metabolism Laboratory, Department of Food Science and Nutrition, Faculty of Food Engineering, University of Campinas, Campinas 13083-862, SP, Brazil

**Keywords:** marolo, Cerrado fruit, health benefits, biological activities, antioxidant, anti-inflammatory, phytochemical composition, phenolic compounds, alkaloids, annonaceous acetogenins

## Abstract

Araticum (*Annona crassiflora* Mart.) is a native and endemic species to Brazilian Cerrado whose fruits have high sensorial, nutritional, bioactive, and economic potential. Its use in local folk medicine, associated with recent scientific findings, has attracted growing interest from different industrial sectors. Therefore, understanding the scientific advances achieved so far and identifying gaps to be filled is essential to direct future studies and transform accumulated knowledge into innovative technologies and products. In this review, we summarize the phytochemical composition, bioactivities, and food products from araticum fruit that have been reported in the scientific literature over the past 10 years. The compiled data showed that araticum fruit parts contain a wide range of bioactive compounds, particularly phenolic compounds, alkaloids, annonaceous acetogenins, carotenoids, phytosterols, and tocols. These phytochemicals contribute to different biological activities verified in araticum fruit extracts/fractions, including antioxidant, anti-inflammatory, anti-Alzheimer, anticancer, antidiabetic, anti-obesity, antidyslipidemic, antinociceptive, hepatoprotective, healing of the cutaneous wound, antibacterial, and insecticide effects. Despite the promising findings, further studies—particularly toxicological (especially, with byproducts), pre-clinical, and clinical trials—must be conducted to confirm these biological effects in humans and assure the safety and well-being of consumers.

## 1. Introduction

Araticum, also popularly known as marolo, articum, ariticum, araticum-do-cerrado, bruto, cabeca-de-negro, cascudo, panã, pinha-do-cerrado, and pasmada, is a fruitful tree, native and endemic to Brazilian Cerrado, belonging to the Annonaceae family [1]. This species is widely distributed along the Cerrado biome areas, covering the states of Minas Gerais, São Paulo, Goiás, Bahia, Mato Grosso, Mato Grosso do Sul, Tocantins, Maranhão, Piauí, Pará, and Federal District [2]. Obtaining the fruits for commercial purposes has been done in an extractive manner from native populations, in areas of the Brazilian Cerrado, due to the absence of commercial plantations. Therefore, araticum plants present high genetic diversity since they come from seeds that have not yet undergone genetic improvements, thus resulting in highly different individuals [3]. Moreover, several factors can affect the genetic diversity of this species, including climatic conditions (e.g., temperature), geographical location, and landscape where the matrix is located (e.g., percentage of forestry cover, water, and urban areas) [4,5].

**Figure 1:**
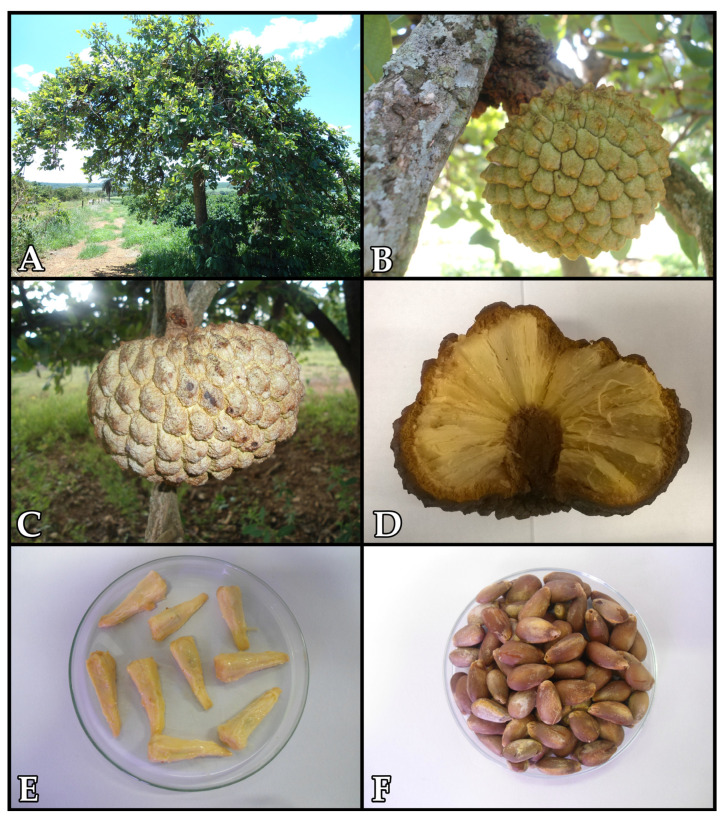
Araticum (*Annona crassiflora* Mart.) (**A**) tree, (**B**) unripe fruit, (**C**) ripe fruit, (**D**) fruit cross-section, (**E**) carpels, and (**F**) seeds. Pictures taken by Henrique Silvano Arruda in natural areas of the Cerrado biome located in the city of Carmo do Paranaíba, Minas Gerais, Brazil (altitude of 1061 meters above sea level at a latitude of 19°00′03″ S and longitude of 46°18′58″ W).

Araticum is a medium-sized tree, reaching 4 to 8 m in height and about 4 m in crown diameter (Figure 1A). The fruit has a subglobose shape, with a green epicarp when unripe and one that is greenish-brown when ripe (Figure 1B,C). The pulp is arranged on a fleshy and tapered receptacle, and it has a color ranging from white to yellowish-pink, a characteristic strong and slightly sweet flavor, and a pleasant smell (Figure 1D). In addition, the pulp is composed of cone-shaped buds (90–190 carpels) that contain a single seed (Figure 1E). The seeds have a flattened obovoid shape and bone consistency with a smooth, opaque, and light brown surface (Figure 1F) [6].

Araticum fruit parts have been used for centuries by folk medicine to treat different pathological conditions. The fruit is used by local and traditional medicine as a tonic and astringent, as well as for treating pain and rheumatism [7,8], whereas seed preparations are reported as antidiarrheic, antitumor, and insecticide agents; inductors of menstruation properties; useful for the treatment of Chagas’ disease; useful against snakebites, skin, and scalp infections [6]. In addition to being used in folk medicine, araticum fruit is highly appreciated by inhabitants of the Cerrado region. This fruit is among the 20 most used species in the regional foodstuffs, being consumed both *in natura* and in the processed form (e.g., juices, jams, ice creams, popsicles, among others), contributing significantly to the intake of essential macro and micronutrients [9]. According to Cardoso et al. [10], the intake of 100 g of araticum pulp provides 1.52 g proteins, 3.50 g lipids, 14.39 g carbohydrates, 0.47 g ash, 95.12 kcal, 6.80 g dietary fiber, 4.98 mg carotenoids, 288.79 RAE (Retinol Activity Equivalent) (vitamin A), 5.23 mg ascorbic acid, 494.04 µg vitamin E, and 27.36 µg folates. Thus, araticum consumption could be a key tool to mitigate food insecurity, malnutrition, and risk for malnutrition-related disorders.

In addition to the attractive sensory attributes and significant nutritional properties of araticum fruit pulp, recent studies have shown that both the edible fruit part (pulp) and its byproducts (peel and seeds) contain several bioactive compounds that exert important biological activities [6]. However, this information is sometimes too dispersed and out-of-date in the specialized literature, making it difficult to understand the scientific advances achieved so far and to identify the gaps to be filled by future research. Therefore, this comprehensive review was designed to summarize the most recent scientific data found in the literature on the phytochemical composition, bioactive properties, and developed food products from araticum fruit to compile up-to-date information that will help clarify the advances made so far and the gaps to be filled by future studies. Thus, this review can be a reference material to support other researchers in conducting future studies on araticum fruit.

## 2. Search Strategy and Studies Selection

In the current comprehensive review study, electronic searches were carried out using the main repositories of the world’s scientific data (Scopus, Google Scholar, Science Direct, Web of Science, and PubMed databases) to identify relevant studies published in scientific journals in the last decade (2013 to the present). We used the following terms to perform our bibliographic research: “Araticum” OR “*Annona crassiflora*”. The abovementioned terms were searched on the article title, abstract, and keywords. The search was not restricted to any specific language and/or journal impact factor. The studies that met the search criteria were selected for full-text review. Theses, editorials, communications, and conference abstracts were excluded. The inclusion criteria were studies that reported results concerning: (1) fruit parts, (2) phytochemical composition, (3) biological properties, and (4) food and beverage products.

## 3. Bioactive Compounds Found in Araticum Fruit Parts

Bioactive compounds are naturally occurring chemical substances found in plants, animals, and microorganisms that have specific biological activities and can exert a wide spectrum of beneficial effects on human health, including antioxidant, anti-inflammatory, antimicrobial, anti-aging, and anticarcinogenic effects. They are classified into diverse classes according to chemical structure, namely phenolic compounds, alkaloids, carotenoids, tocols, phytosterols, organosulfur compounds, polysaccharides, amino acids, and peptides, among others [11,12]. As shown in Table 1, bioactive compounds such as phenolic compounds, alkaloids, annonaceous acetogenins, carotenoids, phytosterols, and tocols were observed in the pulp, peel, and seed of araticum fruit, and they will be discussed in more detail throughout this section.

### 3.1. Phenolic Compounds

Phenolic compounds are an important class of phytochemicals formed from secondary plant metabolites containing hydroxyl (−OH) substituents on an aromatic hydrocarbon chain. This class of bioactive compounds shows a large diversity of structures, including rather simple molecules (phenolic acids) and polyphenols such as stilbenes, flavonoids, lignans, and tannins, that can be found free or associated with carbohydrates, lipids, cell wall components, amines, and organic acids [34]. Phenolic compounds have multiple biochemical actions as antioxidant agents, and they are involved in the modulation of signaling pathways, gene expression, and the modification of epigenetic changes mainly related to chronic non-communicable diseases (e.g., metabolic syndromes, cancers, and neurodegenerative diseases) [35]. Therefore, phenolic compounds can be alternative or complementary tools for the prevention or management of non-communicable chronic diseases.

**Figure 2:**
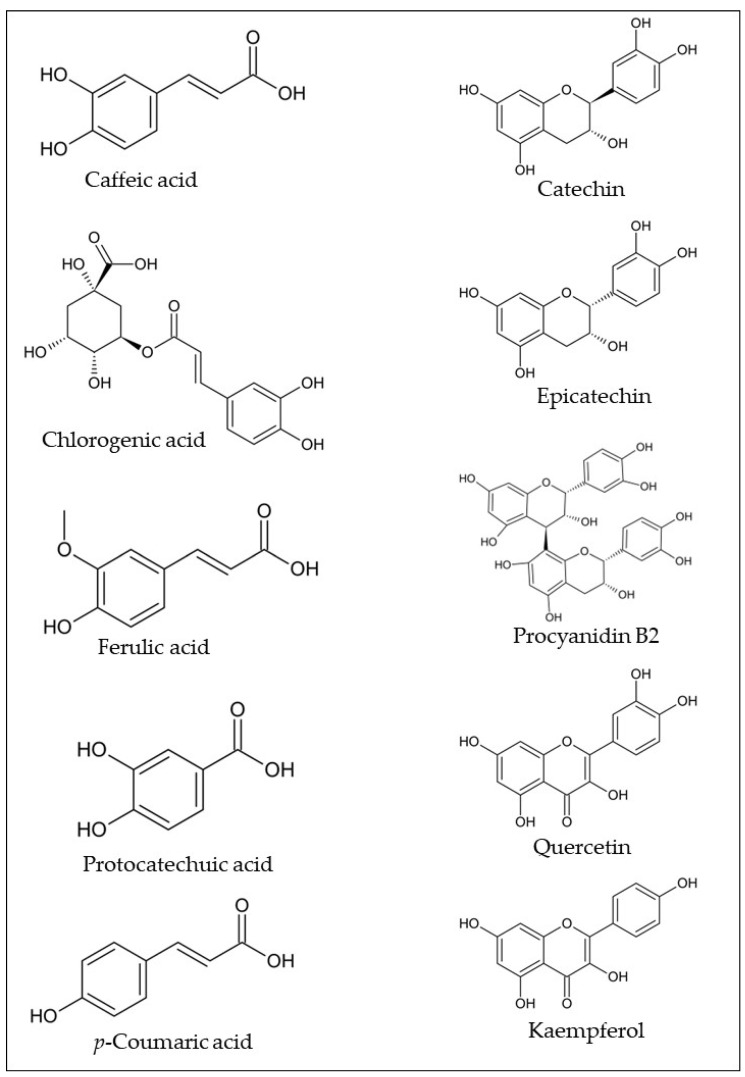
Chemical structures of the most common phenolic compounds found in araticum fruit. Own authorship created by ChemSketch software.

Phenolic compounds are the most studied bioactive compounds class in the araticum fruit parts. As can be seen in Table 1, several phenolic compounds have been identified in araticum fruit, with the most common being caffeic acid, chlorogenic acid, ferulic acid, protocatechuic acid, *p*-coumaric acid, catechin, epicatechin, procyanidin B2, quercetin, kaempferol, and their derivatives (Figure 2). Sophisticated analytical techniques, including high-performance liquid chromatography coupled with diode array detector (HPLC-DAD), high-performance liquid chromatography coupled with mass spectrometry (HPLC-MS), and paper spray ionization mass spectrometry (PS-MS), have been used for their identification and quantification.

Overall, the profile of phenolic compounds from the different parts of the araticum fruit (pulp, peel, and seeds) is diverse. As shown in Table 1, several phenolic compounds have been identified in the pulp (derivatives of 4-hydroxybenzoic acid, *p*-coumaric acid, ferulic acid, gallic acid, sinapic acid, caffeic acid, (epi)catechin, quercetin, kaempferol, rutin, tangeretin, syringic acid, apigenin, and naringenin), peel (derivatives of syringic acid, ferulic acid, protocatechuic acid, hydroxybenzoic acid, caffeic acid, vanillic acid, chlorogenic acid, *p*-coumaric acid, (epi)catechin, kuwanon G, quercetin, kaempferol, apigenin, tangeretin, neocarthamin, isorharmnetin, luteolin, naringenin, vanillin, and lariciresinol), and seeds (derivatives of 4-hydroxybenzoic acid, gallic acid, chlorogenic acid, protocatechuic acid, caffeic acid, *p*-coumaric acid, synaptic acid, ferulic acid, trans-cinnamic acid, catechin, epicatechin, kaempferol, vanillin, quercetin, rutin, naringenin, and tangerentin). Arruda et al. [16] carried out an in-depth exploratory study that allowed for the identification of 112 phenolic compounds (73 flavonoids, 33 phenolic acids, and 6 other phenolics) in a hydroethanolic extract (50% ethanol) from araticum peel.

In general, the content of phenolic compounds can diverge according to fruit part (pulp, peel, and seeds), ripening stage, edaphoclimatic conditions, and storage conditions, as well as the extraction procedures and conditions (e.g., composition and polarity of the solvent, time and temperature of extraction, pH, number of re-extractions, particle size, etc.) and extraction methods (e.g., water bath shaker, maceration, ultrasound, etc.). For example, Ramos et al. [24], evaluating pulp, peel, and seeds of araticum fruit obtained from different regions, found variations of 2-fold, 2.5-fold, and 3.5-fold in the total phenolic content, respectively. In the same study, the araticum peel showed the highest content of total phenolics (837.53–1926.56 mg GAE/100 g), followed by the seeds (358.28–1186.07 mg GAE/100 g) and pulp (480.81–1007.62 mg GAE/100 g). Similarly, Arruda et al. [25] found the highest total phenolic contents in the araticum peel, followed by the pulp and the seeds. Furthermore, Arruda et al. [16] observed a 2-fold increase in total phenolic content from araticum peel when optimizing the ultrasonic power and process time. Another study by Arruda et al. [36] demonstrated that optimization extraction conditions (solvent composition, temperature, and extraction time) increase the total phenolic content extracted from araticum pulp 3-fold.

Regarding the quantification of phenolic compounds, Guimarães et al. [15] observed that catechin (16.79 mg/100 g fw) and gallic acid (1.89 mg/100 g fw) were the major compounds in araticum pulp, followed by amounts lower than 0.55 mg/100 g fw for other phenolic acids (chlorogenic acid, caffeic acid, *p*-coumaric acid, ferulic acid, trans-cinnamic acid, *m*-coumaric acid, and *o*-coumaric acid) and flavonoids (quercetin, vanillin, and rutin). Besides that, Arruda et al. [25] quantified 10 phenolic compounds, namely catechin (768.42 µg/g dw), epicatechin (661.81 µg/g dw), caffeic acid (124.31 µg/g dw), protocatechuic acid (97.92 µg/g dw), ferulic acid (53.71 µg/g dw), chlorogenic acid (43.45 µg/g dw), gentisic acid (14.00 µg/g dw), *p*-coumaric acid (11.86 µg/g dw), rutin (9.31 µg/g dw), and quercetin (7.80 µg/g dw), in araticum pulp.

There has been increasing interest in the utilization of araticum fruit byproducts (peel and seed). Arruda et al. [16] found 14 phenolic compounds in the peel, namely epicatechin (136.47 µg/g dw), rutin (58.53 µg/g dw), chlorogenic acid (16.83 µg/g dw), catechin (15.23 µg/g dw), ferulic acid (10.96 µg/g dw), vicenin-2 (2.60 µg/g dw), vanillin (3.47 µg/g dw), naringenin (2.15 µg/g dw), protocatechuic acid (1.64 µg/g dw), caffeic acid (1.22 µg/g dw), luteolin (0.91 µg/g dw), *p*-coumaric acid (0.49 µg/g dw), 4-hydroxybenzoic acid (0.49 µg/g dw), and vitexin (0.18 µg/g dw). Additionally, Prado et al. [23] showed the presence of 12 phenolic compounds in the peel, such as epicatechin (6221.63 µg/g extract), catechin (579.40 µg/g extract), chlorogenic acid (305.15 µg/g extract), rutin (133.31 µg/g extract), *p*-coumaric acid (14.97 µg/g extract), and traces of quercetin, naringenin, protocatechuic acid, 4-hydroxybenzoic acid, vanillic acid, caffeic acid, and ferulic acid. Moreover, Menezes et al. [20] quantified 11 phenolic compounds in araticum seeds, namely *o*-coumaric acid (3822.5 mg/kg dw), rutin (2209.4 mg/kg dw), *p*-coumaric acid (188.5 mg/kg dw), gallic acid (135.6 mg/kg dw), trans-cinnamic acid (102.6 mg/kg dw), quercetin (83.5 mg/kg dw), ferulic acid (63.9 mg/kg dw), caffeic acid (40.5 mg/kg dw), catechin (35.1 mg/kg dw), chlorogenic acid (14.7 mg/kg dw), and vanillin (3.1 mg/kg dw). Furthermore, Formagio et al. [21] observed the presence of rutin (493 mg/mL), caffeic acid (302 mg/mL), sinapic acid (248 mg/mL), ferulic acid (176 µg/mL), and *p*-coumaric acid (106 µg/mL). Although there is a variation in the content of phenolic compounds due to different ways of obtaining the extract and different analytical methods, as well as other conditions described above, which may influence their content, catechin, epicatechin, rutin, chlorogenic acid, and caffeic acid appear to be the major phenolic compounds found in pulp and peel, whereas seed shows coumaric acid, caffeic acid, rutin, catechin, and epicatechin as its main phenolics. Recent studies have reported the ability of phenolic-rich extracts obtained from araticum fruit to exert beneficial effects on human health, including antitumor, antioxidant, anti-inflammatory, antimicrobial, antihypertensive, and hepatoprotective properties (see Section 4 for more details). Therefore, this fruit offers a wide spectrum of technological applications in the area of functional foods, pharmaceuticals, and cosmetics.

### 3.2. Alkaloids

Alkaloids are a huge class of natural compounds commonly found in the Annonaceae family [37]. This class of secondary metabolites is grouped under heterocyclic and non-heterocyclic compounds based on the position of the nitrogen atom in their chemical structure [38]. Alkaloids are considered bioactive compounds capable of exerting multiple biological activities, such as antioxidant, antidiabetic, anti-inflammatory, antimicrobial, antitumoral, anti-hypertensive, antidiarrheal, antimalarial, and antidiabetic activities [6,38,39]. Additionally, there are already several drugs available on the market produced from natural plant alkaloids [40].

**Figure 3:**
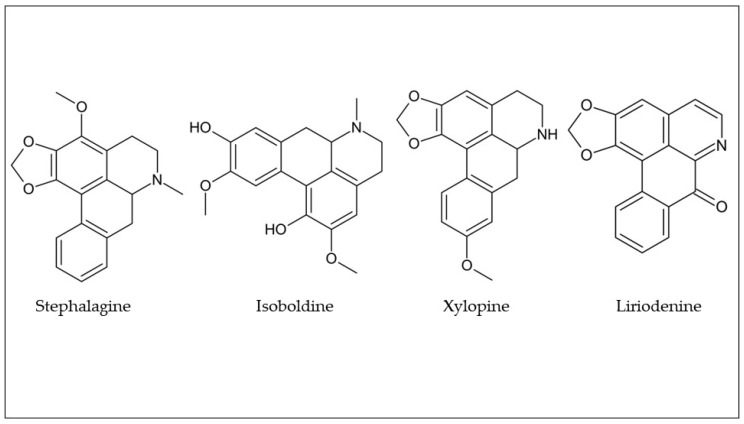
Chemical structures of the most common alkaloids found in araticum fruit. Own authorship created by ChemSketch software.

As can be seen in Table 1, several alkaloids have been found in araticum pulp and peel. However, the most frequently found alkaloids were stephalagine, isoboldine, xylopine, and liriodenine (Figure 3). The stephalagine was purified by semi-preparative HPLC on RP-C_18_ and structurally characterized by high-resolution mass spectrometry, with electrospray ionization (HR-ESI-MS) and nuclear magnetic resonance (NMR) techniques, for the first time by Pereira et al. [29]. Additionally, recent studies have reported the presence of stephalagine in araticum peel and pulp [14,26,27,28,30], indicating that this compound is present in more than a fraction of the fruit. Stephalagine, an aporphine alkaloid, has demonstrated anti-obesity, antinociceptive, and anti-edematogenic effects, suggesting its use as a new potential drug [28,29]. In addition to stephalagine, other alkaloids, such as xylopine, romucosine, asimilobine, roemerine, nornuciferine, *N*-methylcoclaurine, guattescidine, actinodaphnine, isoboldine, and *N*-methyllaurotetanine, have been identified in araticum pulp [14,22]. Several studies demonstrated the presence of liriodenine, atherospermidine, isopiline, isoboldine, isocorydine, anonaine, xylopine, nuciferine, liriodenine, atherospermidine, reticuline, roemerine, nornuciferine, *N*-methylcoclaurine, guattescidine, actinodaphnine, and *N*-methyllaurotetanine in the araticum peel [22,26,30]. As mentioned above, there is a significant interest in isolating and purifying alkaloids from araticum pulp and its byproduct (peel), which can serve as potential sources for obtaining alkaloids for the pharmaceutical industry.

### 3.3. Annonaceous Acetogenins

Annonaceous acetogenins constitute a group of powerful bioactive substances derived from long-chain fatty acids (C35-C37) by polyketide pathways found exclusively in plants of the Annonaceae family. They are commonly characterized by a combination of fatty acids, with a 2-propanol unit at C-2 that forms a methyl-substituted α,β-unsaturated γ-lactone [41,42]. Annonaceous acetogenins have attracted significant scientific interest for the last decade because of their biological activities such as antiparasitic, immunosuppressive, neurotoxic, and pesticidal properties [42]. Beyond that, these compounds appear to be powerful against cancer [6,42,43]. However, chronic exposure to annonaceous acetogenins can potentiate neural damage in the body; thus, moderate consumption of these compounds is recommended [43]. 

**Figure 4:**
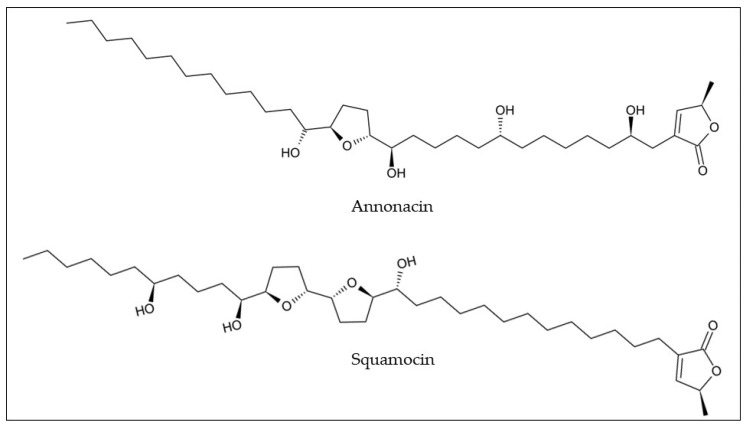
Chemical structures of the most common annonaceous acetogenins found in araticum fruit. Own authorship created by ChemSketch software.

Despite their biological benefits, only five studies have reported annonaceous acetogenin composition in araticum fruits (Table 1). The methanolic extract, from araticum pulp and seeds, revealed the presence of two annonaceous acetogenins, namely annonacin (0.33 and 5.90 mg/g dw) and squamocin (0.40 and 142 mg/g dw), respectively (Figure 4) [31]. The extraction procedure and chromatographic analyses were different for each study, which allowed for the identification of different compounds from the pulp, peel, and seed fractions of the araticum fruit. Arruda et al. [16] investigated the profile of hydroethanolic extract from araticum peel, by high-performance liquid chromatography coupled with mass spectrometry with an electrospray ionization source (HPLC-ESI-MS), and found 21 annonaceous acetogenins. Ramos et al. [22] tentatively identified 17, 9, and 22 annonaceous acetogenins in the ethanolic extracts from pulp, peel, and seeds, respectively, by paper spray ionization mass spectrometry analysis (PS-MS). As reported above, in recent years, there has been an increase in interest in discovering annonaceous acetogenins present in araticum fruit because these metabolites possess a broad spectrum of biological activities and are recognized as anticancer agents. However, further studies are needed regarding their quantification and identification in araticum fruit parts.

### 3.4. Carotenoids

Carotenoids are considered a very diverse group of natural liposoluble pigments responsible for the red, orange, and yellow colors synthesized by plants, microorganisms, and vertebrates [44]. This compound group is comprised of a large class of isoprenoid compounds characterized by a backbone with 40 carbon atoms, the possession of a centrally located conjugated double-bond system, and an ability to carry cyclic or acyclic end groups. This system serves as the chromophore and allows these compounds to absorb wavelengths in the visible spectrum (400–550 nm) [44,45]. Carotenoids are involved in a series of biochemical activities with high antioxidant activity, can display a prebiotic-like effect, and some of them present pro-vitamin A activities. Additionally, there is an association between carotenoid intake and reduced risk of several diseases, especially chronic non-communicable diseases [46].

**Figure 5:**
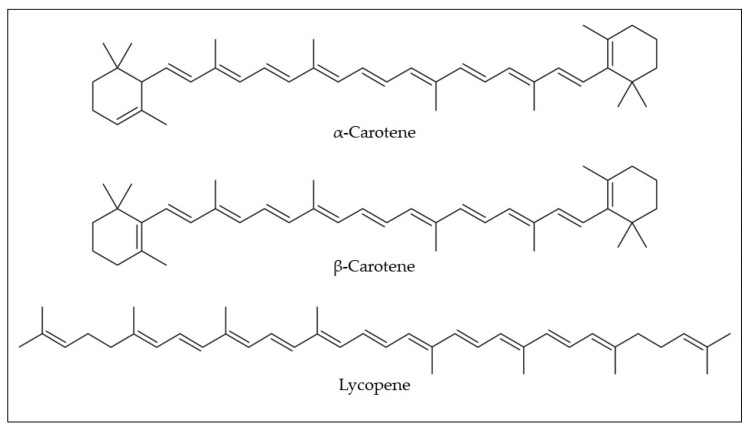
Chemical structures of the most common carotenoids found in araticum fruit. Own authorship created by ChemSketch software.

Carotenoids have been identified and quantified in araticum pulp by high-performance liquid chromatography coupled with the diode array detector method (HPLC-DAD) (Table 1). In the araticum pulp, a low quantity of lycopene (0.02 mg/100 g fw) could be found, while α and β-carotene (2.98 and 1.97 mg/100 g) were its major carotenoids (Figure 5) [10]. Silva et al. [32] found different isoforms of carotenes in araticum pulp, namely all-trans-α-carotene (1.55–1.98 mg/100 g fw) and all-trans-β-carotene (0.86–1.58 mg/100 g fw). As mentioned above, carotenoids exert an important nutritional role, as they present pro-vitamin A activity (e.g., α and β-carotene). Additionally, these compounds are essential for eye health because some pro-vitamin A carotenoids (e.g., α-carotene, β-carotene, γ-carotene, and β-cryptoxanthin) are endogenously converted into retinoids [44]. Although there are few reports on the content and composition of carotenoids in araticum fruit, the literature is consistent in mentioning that the consumption of 100 g of the araticum pulp is enough to reach the recommended daily intake of vitamin A [6]. Thus, *in natura* consumption of this fruit could be an important tool to reduce food insecurity, malnutrition, and risk for several diseases.

### 3.5. Phytosterols

Phytosterols are plant-derived sterols that are structurally similar to cholesterol; however, they differ by the presence of one or two methyl or ethyl groups in the molecule’s side chain [47]. These bioactive compounds can be found in vegetable oils, nuts, and seeds, mainly in the form of sitosterol, campesterol, and stigmasterol [48]. Phytosterols have important roles in the pharmaceutical, medicine, food, and nutrition areas due to their high nutritional value, potent bioactivity, and multiple medicinal effects, such as lowering blood cholesterol levels, anti-inflammatory, antitumoral, antimicrobial, and antioxidant properties, as well as others. [6,48].

**Figure 6:**
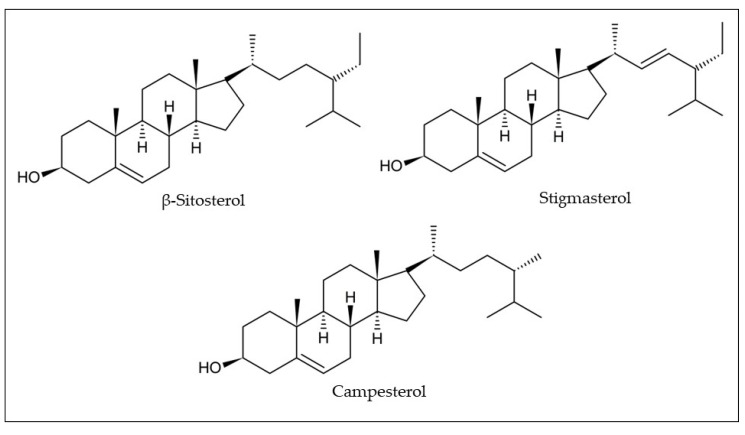
Chemical structures of the most common phytosterols found in araticum fruit. Own authorship created by ChemSketch software.

Phytosterols have been identified and quantified in araticum seeds by gas chromatography with the flame ionization detection method (GC-FID) (Table 1). Luzia and Jorge [33] showed that the seed lipid fraction contains 683.59 mg/kg of total phytosterols, namely β-sitosterol (300.02 mg/kg), campesterol (204.32 mg/kg), and stigmasterol (179.25 mg/kg) (Figure 6) [33]. Although the araticum seeds showed to be a promising source of obtaining phytosterols, little is still known about their phytosterol composition and their real contributions to health benefits.

### 3.6. Tocols

Tocols are a class of bioactive compounds present in various foods, predominantly in fruits and plant seeds [49]. Tocols can be divided into two major groups: tocopherols and tocotrienols, which are distinguished by their chemical structure. Tocopherols (α, β, γ, and δ) contain a chromanol ring and a 16-carbon phytyl side chain in their structure, differing one from the other by position and number of methylation, while tocotrienols differ from tocopherols by possessing double bonds in the 16-carbon side chain at the positions of 3′-, 7′-, and 11′ [49,50]. These bioactive compounds are vitamin E homologs and, therefore, are considered beneficial in the prevention of different pathological conditions, acting as lipid-soluble antioxidants, antihypertensive, neuroprotective, hypolipidemic, antitumoral, and anti-inflammatory materials [6,50].

**Figure 7:**
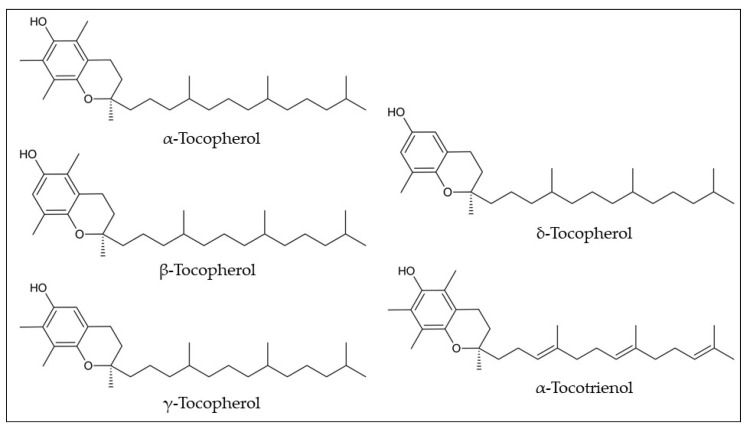
Chemical structures of the most common tocols found in araticum fruit. Own authorship created by ChemSketch software.

Despite their biological benefits, only two studies have reported the tocols’ composition in araticum fruits in the last decade (Table 1). The tocopherol isomers were quantified in the araticum seeds by high-performance liquid chromatography coupled with a fluorescence detector (HPLC-FLD), totalizing 138.90 mg/kg, namely α-tocopherol (12.02 mg/kg dw), β-tocopherol (3.30 mg/kg dw), γ-tocopherol (123.42 mg/kg dw), and δ-tocopherol (0.16 mg/kg dw) (Figure 7) [33]. Cardoso et al. [10] found two tocols in the pulp, with α-tocotrienol being the major tocol (332.94 μg/100 g) followed by α-tocopherol (163.11 μg/100 g), resulting in a total amount of 494.04 μg/100 g of tocols. The recommended daily intake of vitamin E is 15 mg. Therefore, neither the seeds nor the pulp can be considered a source of vitamin E. α-Tocopherol is the only one that meets human requirements for vitamin E, based on the concept that this form is the preferable retained form by the body, and reverses the symptoms of human deficiency. However, the other isomers may contribute to the liposoluble antioxidant function [49]. Thus, the higher content of the γ-tocopherol isomer in the seed oil and α-tocotrienol in the pulp could contribute significantly to this activity.

## 4. Biological Activities Reported in Araticum Fruit Parts

The presence of several bioactive compounds in araticum fruit parts (as summarized above) can contribute to a wide variety of beneficial effects on human health and other bioactivities. In addition to being used as a food source, araticum fruits have been employed for centuries in folk medicine to combat various diseases. Hence, numerous studies have been conducted in recent years to examine the biological activities of araticum fruit parts. Several bioactivities have been reported in extracts/isolated compounds from araticum fruit, as described in the sections below (Table 2).

### 4.1. Antioxidant and Anti-Inflammatory Activities

Uncontrolled and excessive oxidative damage and inflammation are associated with the onset and progression of several non-communicable chronic diseases such as neurodegenerative diseases, cancers, and metabolic syndromes [67]. *In vitro* and *in vivo* studies have shown that extracts obtained from different parts of araticum fruits (pulp, peel, and seeds) present antioxidant and/or anti-inflammatory activities (see Table 2).

A series of studies have reported the antioxidant activity of several extracts and fractions obtained from the pulp [13,14,15,24,25,36,51,52], peel [16,17,18,23,24,25], and seeds [23,24,25,33,53] of araticum fruit through different *in vitro* antioxidant assays, including DPPH, TEAC, ORAC, FRAP, β-carotene/linoleic acid system, TBARS, phosphomolybdenum complex, and reducing power. As can be seen in Table 1, phenolic compounds are the main bioactive compounds reported in these extracts/fractions from araticum fruit parts. In *in vitro* systems, phenolic compounds can perform their antioxidant effects by inhibiting/scavenging free radicals and reactive species, chelating transition metals, and donating hydrogen atoms and/or electrons [16].

Studies using cellular and animal models have proven the *in vitro* antioxidant potential of extracts and fractions obtained from araticum fruit parts. Justino et al. [18] verified that the procyanidin B-rich fraction (0.1–10 μg/mL) and ethyl acetate fraction (1–10 μg/mL) from araticum peel were able to reduce zymosan-induced ROS production in macrophages with no cytotoxicity at 10 μg/mL. Moreover, both the procyanidin B-rich fraction and ethyl acetate fraction (0.5–50 μg/mL), significantly and dose-dependently, reduced Fe^2+^-ascorbate-induced lipid peroxidation and increased total antioxidant capacity in rats’ liver. Lucas dos Santos et al. [54] demonstrated that the aqueous extract from araticum pulp (1 mg/mL) reduced juglone-induced oxidative stress in wild-type (N2) strains of *Caenorhabditis elegans*, improving worm survival rate (32.2% survival) when compared to the control group (5.5% survival). Ramos et al. [19] observed that the oral pretreatment of Triton WR-1339-induced hyperlipidemic mice, for 12 days with an ethanolic extract from araticum peel and its ethyl acetate fraction (10–100 mg/kg bw), was able to attenuate hyperlipidemia-induced oxidative stress in the mice liver by reducing hepatic lipid peroxidation and protein carbonylation, maintaining hepatic total thiol content, restoring hepatic GSH levels, and increasing hepatic antioxidant enzyme activities (SOD, CAT, GPx, GR, and G6PD). Likewise, Justino et al. [55] verified an improved hepatic antioxidant status of streptozotocin-induced diabetic rats receiving a phenolic-rich fraction from araticum peel (25–100 mg/kg bw) for 30 days. Oral administration of this phenolic-rich fraction counteracted the hyperglycemia-induced oxidative stress in the rat liver by reducing hepatic lipid peroxidation, protein carbonylation and nitration, iNOS expression, and the overexpression and activity of some antioxidant enzymes (GPx, SOD, and CAT), besides increasing hepatic total antioxidant activity, GSH content, and GR activity and expression. The *in vivo* antioxidant effects observed in these studies are attributed, at least in part, to the presence of phenolic compounds that can scavenge reactive species, stimulate the endogenous antioxidant defense system, upregulate the expression of endogenous antioxidant molecules and enzymes, and downregulate the expression of endogenous pro-oxidant metabolites and enzymes [67].

High exposure to exogenous sources of reactive species or their endogenous overproduction can trigger inflammatory processes through the activation of nuclear factors (e.g., NF-κB) that initiate the transcription of genes related to inflammation, increasing the production of pro-inflammatory mediators, especially TNF-α and IL-1β cytokines, CXCL1/KC and CXCL2/MIP-2 chemokines, and lipid mediator leukotriene B4, among others [34]. In addition to antioxidant effects, some extracts, fractions, or isolated compounds from araticum fruit, particularly from the peel, also presented anti-inflammatory activity (Table 2). Daily intake of whole araticum pulp (3.214 mL/kg bw) for 30 days promoted a significant reduction in the total leukocytes of rats as compared to the control group [57]. Justino et al. [56] evaluated the effect of different ethanolic extract fractions from araticum peel on pro-inflammatory mediators’ production in an LPS-induced macrophage model. Only phenolic-rich fractions (ethyl acetate (0.1 and 0.3 µg/mL) and *n*-butanol (1 µg/mL) fractions) reduced LPS-stimulated IL-6 and NO production. Topical treatment of wounds induced on the back of mice for 7–21 days with an ointment containing 4% of a phenolic-rich fraction from araticum peel was able to attenuate local inflammation by inhibiting MPO and NAG activities that reduce the activation of neutrophils and macrophages in cutaneous wounds [58,59,60]. Mice pre-treated orally with a phenolic-rich fraction (ethyl acetate fraction) from araticum peel (30 mg/kg bw) showed a reduced formation of CFA-induced paw edema in the acute phase due to the decrease in MPO activity and leukocyte infiltration in the paw tissue [56]. The anti-inflammatory activities of araticum pulp and phenolic-rich fractions from araticum peel are closely related to their phenolic compounds that can modulate endogenous signaling pathways involved in oxidative damage and inflammation, downregulating pro-inflammatory mediators’ expression and, consequently, blocking the triggering of the inflammatory cascade [67].

In addition to phenolic compounds, stephalagine, an alkaloid isolated from araticum peel, was also able to alleviate the inflammatory process in animal trials. Oral administration of stephalagine (1 mg/kg bw) mitigated inflammation in mice by reducing capsaicin-induced paw edema [28]. Similarly, Santos et al. [27] reported that monosodium urate crystal-induced gout mice, treated orally with stephalagine (1 mg/kg bw), had attenuated inflammation signs, including a reduction in articular edema, MPO activity, IL-1β level, and neutrophil infiltration in joints. Together, these findings support the anti-inflammatory effects of the stephalagine isolated from araticum peel and suggest that this activity is triggered, at least in part, by suppressing pro-inflammatory cytokines and neutrophil infiltration.

### 4.2. Anti-Alzheimer Activity

Neurodegenerative diseases are a group of brain disorders that constitute a significant global public health concern, as they compromise individuals’ life quality and cause an enormous mental and economic burden on caregivers [67]. It is estimated that around 50 million people are living with some form of dementia, and the number of affected individuals should reach 152 million by 2050. Alzheimer’s disease is the most common dementia form, accounting for 60–70% of all cases [68]. In Alzheimer’s disease, cholinergic neurotransmitter levels, especially acetylcholine, are reduced in the synaptic cleft, compromising the synaptic signaling pathways and, thus, contributing to cognitive and motor dysfunctions [69]. Therefore, cholinesterase enzyme inhibitors may be promising tools to alleviate neurological disorders by reducing the degradation of neurotransmitters, especially acetylcholine. Formagio et al. [21] reported the *in vitro* anticholinesterase activity of methanolic extract from araticum seeds. This extract inhibited 45% of acetylcholinesterase (AChE) activity at 1.5 mg/mL. Likewise, Barbosa et al. [26] studied the effect of ethanolic extract from araticum peel, its alkaloidal fraction (dichloromethane fraction), and isolated alkaloids against AChE and butyrylcholinesterase (BChE) enzymes. Ethanolic extract and alkaloidal fraction displayed a low anticholinesterase activity, whereas isolated alkaloids (stephalagine, liriodenine, and atherospermidine) strongly inhibited the AChE and BChE enzymes. Stephalagine and liriodenine presented remarkable inhibitory activity against BChE, showing IC_50_ values (32.7 and 46.2 µg/mL, respectively) lower than or similar to those of the positive control galantamine (IC_50_ value of 47.0 µg/mL). On the other hand, liriodenine showed the highest AChE inhibitory activity with an IC_50_ value (2.7 µg/mL) very close to the positive control galantamine (IC_50_ value of 1.5 µg/mL). Phenolic compounds and alkaloids can interact with active sites of cholinesterase enzymes (e.g., enzyme catalytic sites), delaying or inhibiting their catalytic activities [26,70]. In another study, Lucas dos Santos et al. [54] verified that the treatment with aqueous extract from araticum pulp (1 mg/mL) can reduce the juglone-induced oxidative stress in wild-type (N2) strains of *Caenorhabditis elegans* (enhanced survival rates (32.2% survival) when compared to the control group (5.5% survival)) and paralysis in CL2006 strains of *C. elegans* expressing the Aβ1–42 peptide in muscle tissue (decreased the mean of worms paralyzed 10 h after treatment by 13.4%). The authors have not identified the bioactive compounds present in this extract, but the literature reports that araticum pulp extracts are rich in phenolic compounds (Table 1). Phenolic compounds can exert their anti-Alzheimer effect through several mechanisms (for details, consult the literature review by Arruda et al. [67]).

### 4.3. Anticancer Activity

Cancer is a group of disorders characterized by continuous and excessive cell growth, resulting in an abnormal cell mass. Cancer represents a significant global public health problem, as it affects around 11 million people worldwide each year, accounting for 1 in 6 deaths annually [6,71]. Recent studies have shown the anticarcinogenic potential of extracts and bioactive compound-rich fractions from araticum fruits against different tumor cell lines (Table 2).

Justino et al. [30] have found that the alkaloid and acetogenin-rich fraction (dichloromethane fraction) from araticum peel presented significant antiproliferative activity against liver cancer cells (HEP-G2 cells). This fraction, at 50 µg/mL, reduced HepG2 cell viability, proliferation, and migration without presenting toxicity effects against fibroblast (NIH/3T3) and human peripheral blood mononuclear cells. Complementary assays demonstrated that the HepG2 cells’ growth-inhibitory effect of alkaloid and acetogenin-rich fraction can be triggered, at least in part, by downregulating PCNA and EGFR expression, upregulating ROS production, and inducing mobilization of intracellular Ca^2+^ in the HepG2 cells. Prado et al. [23] investigated the cytostatic potential of methanol-acetone-water (7:7:6, *v*/*v*/*v*) extracts from araticum peel and seeds against eight human cancer cell lines. Neither extract was able to inhibit only NCI-H460 cells (lung, non-small cell type). The seed extract was the most active against all human cancer cell lines tested with total growth inhibition (TGI) values varying from 5.36–76.77 μg/mL, whereas TGI values were between 37.64 and 230.12 μg/mL for peel extract. Cancer cell lines NCI-ADR/RES (ovary with multidrug resistance phenotype), PC-3 (prostate), and OVCAR-3 (ovary) showed the highest susceptibilities to seed extract (TGI values of 5.36, 16.60, and 20.93 µg/mL, respectively). The peel extract was particularly active against the UA251 cells (glioblastoma), whose TGI value was 37.64 μg/mL. This work provided evidence that the seed extract is a promising antiproliferative agent against NCI-ADR/RES cells (ovary with multidrug resistance phenotype), as it presented a TGI value (5.36 µg/mL) significantly lower than the positive control doxorubicin (20.92 µg/mL). Similarly, Formagio et al. [21] conducted a study to evaluate the antiproliferative activity of methanolic extract from araticum seeds against ten human cancer cell lines. This extract showed potent antitumor activity against all human cancer cell lines tested, with GI_50_ values varying from 0.01–8.90 µg/mL. The seed extract was strongly active, in particular, against HT-29 (colon), NCI-H460 (lung, non-small cell type), UA251 (glioblastoma), and NCI-ADR/RES (ovary with multidrug resistance phenotype) cells, showing GI_50_ values of 0.01, 0.04, 0.06, and 0.25 µg/mL, respectively. These GI_50_ values were lower than the positive control doxorubicin (GI_50_ values of 2.20, 0.05, 5.13, and 1.68 µg/mL for HT-29, NCI-H460, UA251, and NCI-ADR/RES cells, respectively). The effect of hydroethanolic extract (70% ethanol) from araticum pulp on the proliferation of six human cancer cell lines was studied by Carvalho et al. [14]. This extract showed significant antiproliferative activity against only UA251 cells (glioblastoma), with a GI_50_ value of 21.34 µg/mL and high tumor cell selectivity, as its GI_50_ value for non-tumor cells (HaCaT) was higher than 100 µg/mL. The antiproliferative potential of araticum fruit can be attributed to the different bioactive compounds present in its extracts, especially annonaceous acetogenins, phenolic compounds, and alkaloids (see Table 1). These compounds can act alone or together, modulating several biochemical pathways involved in the proliferation and death of tumor cells.

### 4.4. Antidiabetic Activity

Diabetes mellitus is a common non-communicable disease that affects millions of people around the world. Diabetes mellitus and its associated complications (e.g., hypertension, cardiovascular disease, obesity, dyslipidemia, atherosclerosis, and chronic kidney disease) are global health issues, as they are among the leading causes of morbidity and mortality. Studies estimate that around 592 million people will be afflicted by this disease by 2035 [72]. Phenolic-rich extracts/fractions obtained from araticum peel have shown promise in managing diabetes mellitus and its complications (Table 2).

Justino et al. [17] investigated the potential of ethanolic extract from araticum peel and its fractions (hexane, dichloromethane, ethyl acetate, *n*-butanol, and aqueous) in inhibiting carbohydrase enzymes (α-amylase and α-glucosidase). This study demonstrated that phenolic-rich fractions (ethyl acetate and *n*-butanol fractions) had the highest carbohydrase inhibitory activity, particularly against α-amylase (IC_50_ values of 4.5 and 1.7 μg/mL for ethyl acetate and *n*-butanol fractions, respectively). Phenolic compounds are effective inhibitors of carbohydrase enzymes due to their ability to bind to the active site of these enzymes or occupy the substrate binding pocket [34].

Persistent hyperglycemia induces irreversible protein glycation, culminating in the formation of advanced glycation end-products (AGEs). These AGEs are involved in the development and progression of different diabetic complications (e.g., nephropathy, retinopathy, and neuropathy) due to their ability to generate reactive species and alter the structures and functions of proteins, lipids, enzymes, and DNA [72]. *In vitro* studies conducted by Justino et al. [17,18] reported that the phenolic-rich fractions from araticum peel inhibited protein glycation. Ethyl acetate and *n*-butanol fractions from araticum peel exhibited the highest protein glycation inhibitory activity by the BSA-fructose model, showing IC_50_ values (14.3 and 16.0 μg/mL, respectively) similar to positive control quercetin (IC_50_ value of 16.6 μg/mL). In addition, these fractions had low cytotoxicity on NIH/3T3 fibroblast cells (cell viability higher than 88.7% at 30 µg/mL) [17]. In another study, Justino et al. [18] verified that procyanidin-rich fraction and ethyl acetate fraction from araticum peel were able to reduce the AGEs production in the BSA-fructose (IC_50_ values of 7.9 and 22.5 µg/mL), BSA-methylglyoxal (IC_50_ values of 128.9 and 228.0 µg/mL), and arginine-methylglyoxal (IC_50_ values of 249.3 and 208.3 µg/mL) models. Moreover, the procyanidin-rich fraction decreased the glycation-induced protein carbonyls formation, glycation-induced protein thiol group oxidation, and AGEs-induced protein crosslinks, while attenuating the decrease in glycated CAT activity. Phenolic compounds present in these fractions can be effective antiglycation agents by suppressing AGEs formation through: the capture of precursors (e.g., dicarbonyl intermediates such as methylglyoxal), preventing them from binding and damaging proteins; the interaction with glucose, making it unavailable to bind to proteins; the inhibition of Schiff’s base and Amadori products formation; and the blockade of AGEs receptors [34]. Moreover, Justino et al. [55] verified that streptozotocin-induced diabetic rats orally treated with a phenolic-rich fraction (*n*-butanol fraction) from araticum peel (100 mg/kg bw for 30 days) had lower glycemia and hepatic oxidative damage/inflammation when compared to the untreated diabetic group. These studies showed that phenolic-rich fractions from araticum peel can be promising therapeutic agents in the management of diabetes mellitus and its associated complications by reducing intestinal carbohydrate digestion and intestinal glucose absorption through the inhibition of key carbohydrate digestive enzymes, by inhibiting AGEs formation, and by attenuating oxidative damage and inflammation.

### 4.5. Anti-Obesity and Antidyslipidemic Activities

Obesity is a major worldwide health concern due to its increasing prevalence. According to World Health Organization, around 39% and 13% of the world’s adult population were overweight and obese, respectively, in 2016 [73]. Obesity is associated with an increased risk of several chronic non-communicable diseases (e.g., cardiovascular diseases, hypertension, type 2 diabetes, dyslipidemia, and cancer) and numerous adverse health effects [74]. Therefore, increasing efforts have been directed toward the search for potential anti-obesity agents. In this context, Pereira et al. [29] evaluated the *in vitro* inhibitory potential of ethanolic extract, its dichloromethane fraction, and stephalagine alkaloid from araticum peel against pancreatic lipase. Stephalagine showed the highest inhibitory activity against pancreatic lipase (IC_50_ value of 8.35 μg/mL), while the ethanolic extract and dichloromethane fraction exhibited lower and similar activity (IC_50_ values of 104.50 and 108.10 μg/mL, respectively). This study showed that the stephalagine has high inhibitory activity against pancreatic lipase and low cytotoxicity on Vero cells (CC_50_ value of 353 μg/mL). On the other hand, Ramos et al. [19] studied the effect of the ethanolic extract from araticum peel and its phenolic-rich fraction (ethyl acetate fraction) on Triton WR-1339-induced hyperlipidemic mice. The pretreatment for 12 days, via orogastric administration of this extract and phenolic-rich fraction, improved serum lipid profile (increased HDL-cholesterol level), reduced lipid hepatic accumulation (decreased hepatic triglycerides and total cholesterol), and attenuated hepatic oxidative stress (reduced lipid peroxidation, protein carbonylation, and thiol groups’ oxidation, while increasing the GSH levels and activity of endogenous antioxidant enzymes (SOD, CAT, GPx, GR, and G6PD)). Furthermore, the histological analyses of mice treated with the extract and phenolic-rich fraction did not show any signs of hepatotoxicity. These antidyslipidemic effects can be attributed to the ability of the phenolic compounds present in this extract/fraction to attenuate oxidative stress, inhibit fat absorption, activate fat metabolism in the liver, promote fatty acid catabolism, and regulate AMP-activated protein kinase pathways. The findings observed in these works suggest the use of phenolic compounds and alkaloids isolated from araticum peel as new potential anti-obesity and antidyslipidemic drugs.

### 4.6. Hepatoprotective Activity

The liver is an essential organ for maintaining the homeostasis of the human body, as it is involved in several biochemical pathways responsible for growth, immunity, nutrient supply, energy provision, reproduction, and xenobiotic elimination. However, hepatotoxic agents (e.g., some viruses, microorganisms, drugs, and metabolites of the organism itself) can trigger a series of liver disorders (e.g., inflammation, fibrosis, and cirrhosis), resulting in about 2 million deaths around the world annually [75,76]. Cellular and animal studies have reported that extracts and fractions obtained from araticum fruit parts are effective and safe hepatoprotective agents (Table 2).

Justino et al. [18] evaluated the hepatoprotective effects of procyanidin B-rich fraction and ethyl acetate fraction from araticum peel on rats’ liver tissue exposed to FeSO_4_ and ascorbic acid. Both fractions (0.5–50 μg/mL) protected rats’ liver tissue against Fe^2+^-ascorbate-induced lipid peroxidation and increased hepatic total antioxidant capacity. The ethanolic extract from araticum peel and its ethyl acetate fraction displayed significant hepatoprotective activity in Triton WR-1339-induced hyperlipidemic mice [19]. The oral pretreatment for 12 days, with this extract or fraction (10–100 mg/kg bw), was able to protect the mice’s livers from hyperlipidemia-induced oxidative stress (decreased hepatic lipid peroxidation, protein carbonylation, and thiol group oxidation; restored hepatic GSH level; increased hepatic antioxidant enzymes activity (SOD, CAT, GPx, GR, and G6PD)) and lipid accumulation (reduced hepatic triglycerides and total cholesterol levels). Similar results were observed in streptozotocin-induced diabetic rats orally treated with a phenolic-rich fraction (*n*-butanol fraction) from araticum peel (25–100 mg /kg bw) for 30 days [55]. This phenolic-rich fraction suppressed the hepatotoxic effects of hyperglycemia, reducing serum liver enzyme activities (ALT, AST, and ALP) and improving some hepatic biochemical parameters (reduced lipid peroxidation, protein carbonylation and nitration, iNOS level, and overexpression and activity of some antioxidant enzymes (GPx, SOD, and CAT) while increased GR activity, GSH content, and total antioxidant activity). The hepatoprotective effect of phenolic-rich extracts/fractions from araticum peel is due to the ability of their phenolic compounds to modulate several liver biochemical responses such as lipid peroxidation reduction, reactive species neutralization, endogenous antioxidant system regulation, DNA repair, and xenobiotic detoxification activation [6,34].

### 4.7. Antinociceptive Activity

Pain is the most common symptom pointed out by patients during medical consultations, and it is associated with several pathological conditions [77]. Pain can be classified as acute or chronic according to its duration. Acute pain is defined as pain lasting up to 3 months, being considered an adaptive signal that prevents danger and guarantees survival. Meanwhile, chronic (or persistent) pain is defined as pain persisting for more than 3 months and it is typically associated with chronic diseases and non-treated medical pathologies, adversely affecting individuals’ physical and mental functioning, productivity, and quality of life [78,79]. As can be seen in Table 2, alkaloids and phenolic compounds present in araticum peel showed analgesic activity in animal models.

Ethanolic extract from araticum peel (30 mg/kg bw), its alkaloidal fraction (dichloromethane fraction at 30 mg/kg bw), and stephalagine alkaloid (0.3 and 1 mg/kg bw), administered orally, exhibited antinociceptive activity in mice by reducing formalin-induced paw licking without affecting the motor performance of the animals [28]. Moreover, this study reported that oral administration of stephalagine (1 mg/kg bw) reduced cinnamaldehyde-, capsaicin-, and formalin-induced paw licking. Similar results were reported by Santos et al. [27] in a monosodium urate crystals-induced gout arthritis mice model. Oral administration of stephalagine (1 mg/kg bw) attenuated the inflammatory process (decreased neutrophil and leukocyte infiltration, MPO activity, and IL-1β level), joint edema development, mechanical allodynia, spontaneous nociception, and cold hypersensitivity without causing any hepatic (maintained the serum ALT and AST activities) or kidney (maintained the serum urea and creatinine levels) damage or possible adverse events (e.g., abnormalities in the diet, sudden changes in body weight, changes in the hair, feces, behavior, and macroscopic anatomy of the mice). According to the authors, the antinociceptive effect of stephalagine can be attributed, at least in part, to its capacity to reduce the activation of channels involved in the transduction and sensitization of primary afferent somatosensory neurons, particularly TRPV1 and TRPA1 channels [27,28]. A phenolic-rich fraction from araticum peel (ethyl acetate fraction) was also able to reverse inflammatory and nociceptive processes in mice [56]. The orally administered phenolic-rich fraction (30 mg/kg bw) decreased glutamate-induced spontaneous nociception (reduced paw licking), CFA-induced early and late hyperalgesia, CFA-induced cold hyperalgesia (reduced paw licking and shaking), CFA-induced inflammation (reduced leukocyte infiltration in the paw tissue and MPO activity), and CFA-induced paw edema without altering the animals’ locomotor activity. Antinociceptive and anti-inflammatory effects of this phenolic-rich fraction from araticum peel can be mediated, at least in part, by its flavonoids and proanthocyanins (see Table 1), which are able to modulate glutamate receptors, reduce oxidative stress and NO production, and downregulate pro-inflammatory cytokine expression and secretion [56].

### 4.8. Healing of Cutaneous Wounds Activity

The skin is the largest organ in the human organism, playing various key functions in the body, including protection against physical aggression and excessive water loss, thermoregulation, secretion, and immunological activity. When the skin suffers any damage, a series of partially overlapping processes are triggered, leading to the reconstruction of the injured area. However, disruption at any phase impedes the sequential healing process, resulting in chronic wounds [58]. *In vitro* and *in vivo* studies have demonstrated the potential of phenolic-rich extracts from araticum fruit in accelerating the wound healing process (Table 2).

Prado et al. [23] verified that a phenolic-rich extract from araticum seeds promoted keratinocyte (HaCaT) migration, leading to 73% of slot closure at 3.6 µg/mL. Animal trials conducted by de Moura et al. [58,59,60] showed that topical application of an ointment, containing 4% of a phenolic-rich fraction from araticum peel, on wounds induced on the back of mice, for 7–21 days, accelerated the cutaneous wound closure by reducing local inflammation (decreased neutrophil and macrophage activation, as well as MPO and NAG activities) and oxidative damage (decreased lipid peroxidation while increased CAT activity), stimulating the profibrogenic process (increased number of mast cells and deposition of types I and III collagen fibers in wounds), promoting keratinocyte and fibroblast proliferation and migration (increased MMP-2 and MMP-9 activities), and improving dermis and epidermis organization during healing. This wound healing effect can be attributed to the ability of phenolic compounds present in these extracts/fractions (see Table 1) in modulating different biochemical pathways involved in the tissue repair process [6].

### 4.9. Antibacterial Activity

Pathogenic bacteria infections are a major cause of morbidity and mortality in the world, causing growing public health concerns worldwide [80]. Hydroethanolic extracts from araticum fruit parts have shown antibacterial effects against human pathogenic bacteria (Table 2).

Silva et al. [61] evaluated the effect of hydroethanolic extract (70% ethanol) from araticum fruit parts (pulp, peel, and seeds) against 60 samples of Oxacillin Resistant *Staphylococcus aureus* (ORSA) isolated from the aerial environment sources (dental care clinic) and *S. aureus* ATCC 6538 (standard strain). The results demonstrated that the seed extract did not have any antibacterial activity against the tested strains of *S. aureus* ATCC 6538. On the other hand, peel and pulp extracts were highly active against *S. aureus* ATCC 6538, showing MIC of 6.25 and 12.5 mg/mL, respectively. All extracts from araticum fruit parts were relatively active against ORSA, where pulp extract showed the highest activity (MIC of 25 mg/mL), followed by peel and seed extracts (MIC of 50 mg/mL). In another study, Stafussa et al. [13] investigated the antibacterial activity of hydroethanolic extract (40% ethanol) from araticum pulp against four potentially pathogenic bacteria. This extract was more active against gram-positive bacteria with MIC of 12.5 and 25 mg/ mL for *B. cereus* and *S. aureus*. The antibacterial activity of these hydroethanolic extracts can be associated with the presence of bioactive compounds, particularly phenolic compounds. Hydroxyl groups present in the chemical structure of these compounds can cause destabilization and permeabilization of the bacteria cell membranes through metal chelation (e.g., iron and zinc) and inhibition of bacteria enzymes, delaying the growth and/or multiplication of these microorganisms [13,34].

### 4.10. Insecticide Activity

Insects and their larvae can cause significant losses in the production and quality of plant crops, and they can also be important vectors of disease-causing viruses transmitted to humans. Currently, insect control has been performed, mainly, through the use of broad-spectrum chemical insecticides. However, the increasing insecticide resistance in insects and concerns for environmental toxicity and human health have accelerated the search for bioinsecticides [6]. Different investigations have demonstrated that extracts from araticum seeds have shown significant activity against some larvae and insects, including disease-carrying insects (e.g., dengue mosquito larvae) and insects/larvae causing crop production losses (e.g., rice stalk stink bug, soybean looper, and brown stink bug) (Table 2).

Costa et al. [62] explored the larvicidal activity of different extracts from araticum seeds and their fractions against third-instar dengue mosquito larvae. Some extracts (hexane, dichloromethane, and methanolic (defatted with hexane) extracts) and hexane fraction were able to kill up to 100% of dengue mosquito larvae at 1 mg/mL. Among the extracts and their fractions tested, methanolic extract (defatted with hexane) was the most active, followed by dichloromethane extract, hexane fraction, and hexane extract (IC_50_ 0.100, 0.185, 0.433, and 0.507 mg/mL, respectively).

Insecticide potential of chloroform–methanol (2:1) extract from araticum seeds was evaluated against rice stalk stink bug nymphs [63], soybean looper eggs, and caterpillars [64]. Krinski and Massaroli [63] verified that topical application of this extract (0.5–8.0%) reduced the mobility and dose-dependently controlled the second-instar rice stalk stink bug nymphs, killing up to 81% of nymphs 120 h after treatment. In another study conducted by the same group, Massaroli et al. [64] investigated the impact of this extract (0.5–8.0%) on different development stages of soybean looper by using two strategies: (1) direct contact and (2) larvae ingestion of treated soybean leaves. The results demonstrated that topical application was the most efficient way to control first-, third-, and fifth-instar caterpillars, reaching up to 93.3% mortality 120 h after application. Moreover, the administration of extract-treated soybean leaves increased the mortality rates for first- and third-instar caterpillars, reducing the number of caterpillars that completed their development.

Studies conducted by Turchen et al. [65] and Silva et al. [66] showed the insecticide activity of methanolic extract from araticum seeds against brown stink bug nymphs and adults. Methanolic extract (0.5–8.0%) killed the third-instar nymphs in a dose-dependent manner [65]. Meanwhile, the spraying of this extract at 2% on a soybean crop was able to reduce the brown stink bug population after 7 days of its application when compared to the control [66].

Although the phytochemicals responsible for the insecticide activity have not been identified in the tested extracts, the authors attribute this effect, particularly, to the presence of annonaceous acetogenins. In fact, these bioactive compounds have been widely reported in different araticum seed extracts, as can be seen in Table 1 and other older works [81,82,83]. Several studies have highlighted the insecticide potential of annonaceous acetogenins against different larvae and insects. Annonaceous acetogenins are powerful mitochondrial inhibitors because they block the respiratory chain at complex I (NADH:ubiquinone oxidoreductase) of the mitochondrial electron transport system which results in ATP deprivation, consequently causing apoptosis [84]. Recent studies have also shown that annonaceous acetogenins can cause damage in the insect midgut epithelium and digestive cells, increasing the expression of genes associated with autophagy induction (e.g., *Atg1* and *Atg8* genes) and decreasing the expression of genes linked with the absorption and transport of water, nutrients, metabolites, and non-electrolytes (e.g., *V-ATPase* and *Aqp4* genes) [85,86].

## 5. Applications of Araticum Fruit in the Foods and Beverages Development

The araticum is a seasonal fruit, with fruiting occurring from January to May, which impairs its availability in the rest of the year. Furthermore, this fruit is highly perishable, making it difficult to preserve the postharvest freshness. For this reason, one way to make it available during the off-season is to diversify the way it is presented to the consumer market through methods that allow its conservation without affecting the sensory and nutritional characteristics [87,88]. 

Araticum fruit presents unique sensory characteristics, such as a lightly sweet pulp with intense flavor, attractive color (ranging from white to yellow), and an exotic aroma, which makes it well accepted by the local population. Thus, this fruit is among the most consumed species in the Cerrado region [25,89]. In addition to the interesting sensory aspects, the araticum fruit has a high nutritional value and is a source of bioactive compounds, being rich in phenolic compounds (phenolic acids and flavonoids), carotenoids, tocopherols, vitamins, dietary fibers, and oligosaccharides [23,89,90].

Physicochemical aspects, such as total titratable acidity (0.51 g citric acid/100 g), pH (4.64), humidity (74.30%), and high levels of total soluble solids (13° Brix) [6,91], allow araticum fruit to be widely used for dessert production, such as sweets (sweet paste, fruit preserves, and milk caramel) and jams, but the fruit can also be used in the fermented milk drinks, juices, yogurt, food bars, bread, and flour (Table 3).

According to Guiné et al. [92], measurements of food preference and acceptance allow an understanding of consumer behavior towards conventional or novel products. Therefore, sensory acceptance is one of the essential aspects in the process of developing new products or product improvement to ensure sensory quality and success in the food products market [90,92]. In this context, several studies showed that the food products developed with araticum pulp showed high sensory acceptance and considerable purchase intention (Table 3), demonstrating that araticum fruit is a promising fruit for producing conventional or innovative healthy products.

The use of the pulp provided numerous nutritional and functional benefits to the food products (Table 3). Jams developed with araticum pulp showed high concentrations of phenolic compounds [93], as well as araticum juices, which presented high phenolic compounds content and increased antioxidant activity [94,95]. In addition, incorporating araticum pulp flour in bread and food bars considerably improved the content of dietary fiber, carotenoids, phenolic compounds, antioxidant activity [96,97,98,99], and vitamin C and minerals levels in the food bars [96,97].

The results demonstrate a wide range of successful uses of araticum pulp in the formulation and development of food products. Although no studies have been found on applying araticum peel and seed for food purposes in the last 10 years, these byproducts are promising sources of bioactive compounds, including phenolic compounds, annonaceous acetogenins, and alkaloids (see Section 3 for more details) that present therapeutic potential [22]. Moreover, Arruda et al. [25] reported that araticum peel had higher phenolic compounds, flavonoids, condensed tannins, and antioxidant activity than the pulp and seed of the fruit, while Menezes et al. [20] found that araticum seeds are good sources of nutritional compounds, such as dietary fiber (351.1 g/kg), proteins (188.7 g/kg), and lipids (311.3 g/kg). Furthermore, the araticum seeds can be a source of oil with a high content of unsaturated fatty acids and with a predominance of oleic and linoleic acids.

These nutritional and biological (as discussed in Section 4) aspects suggest that araticum byproducts, usually discarded, may promote beneficial health effects. Thus, using the araticum peel and seeds can present significant technological advantages in the food and pharmaceutical industries, contributing to the reduction in waste and the generation of value-added products, such as flour, bakery products, food supplements, medicines, cosmetics, and functional ingredients [24,25].

**Table 3:** A summary of studies showing the use of araticum fruit in the development of food products.

Product	Formulation Description	Major Findings	Ref.
Bread	Araticum pulp flour (10 and 20%) in replacing the wheat flour	All bread developed showed high acceptance. Thus, breads made with araticum pulp flour and the control bread obtained average acceptance scores between the hedonic terms “I liked” and “I liked very much” and acceptability indexes above 80%.	[100]
Bread	Araticum pulp flour (10%) replacing refined or whole wheat flour	The addition of araticum pulp flour increased the bread’s antioxidant capacity, favoring its functional potential. The breads developed were well accepted by consumers, with scores above 70%, except for the flavor attribute for whole wheat bread with araticum pulp.	[98]
Bread	Araticum pulp flour (16% + 30% replacing the wheat flour and the water, respectively) and pequi peel flour (2%)	Bread enriched with Cerrado fruits had higher levels of dietary fiber, carotenoids, phenolic compounds, and antioxidant activities than the control bread besides was well-accepted sensorially by children.	[99]
Flour	Araticum pulp	The araticum pulp flour showed high levels of dietary fiber, as well as antioxidant activity, vitamin C, and minerals (potassium, phosphorus, and calcium). Araticum flour demonstrated important technological functional properties, such as good solubility in water and the ability to form an emulsion.	[101]
Fruit preserve	Mixed Brazilian Cerrado using 60% of fruits (araticum, soursop, and passion fruit) added in equal proportions	The mean values of the evaluated sensory attributes ranged from 4 to 8, corresponding between the regions of the hedonic scale “I disliked a little” and “I liked a lot”, respectively. Therefore, the fruit preserves can be considered well-accepted.	[102]
Fruit preserve	Araticum pulp	Preserve formulations with a higher pulp/sugar ratio and concentrations of citric acid resulted in products with higher yields and levels of protein, vitamin C, and fiber.	[103]
Sweet paste	Araticum pulp	All formulations showed good sensory acceptance, with the overall impression, aroma, and flavor scores between 5 (neither liked nor disliked) and 7 (moderately liked), while the purchase intention scores were between 2 (I would probably not buy) and 3 (I would probably buy).	[104]
Sweet paste	Araticum pulp	The sweet pastes developed showed nutritional potential with considerable dietary fiber and calcium contents while being sources of vitamins C and A.	[105]
Sweet paste	Araticum pulp (50 pulp/50 sugar ratio)	The sweet paste with araticum was well accepted by the children, and the hedonic term “I loved” had the highest summation of scores.	[106]
Jam	Mixed Brazilian Cerrado fruit pulp (20% of the araticum, soursop, and sweet passion fruit)	Two jams were thermally processed with or without a vacuum. Both low-calorie mixed jams can be considered with high concentrations of polyphenols.	[93]
Jam	Mixed Brazilian Cerrado fruit pulp (20% of the araticum, soursop, and sweet passion fruit)	The titratable acidity, soluble solids, and total sugar values increased, while the total carotenoids and total phenolics values slightly decreased during storage (180 days). The microbiological analysis indicates that the mold and yeast count in the jams was within the limits recommended by the legislation during storage.	[107]
Jam	Extra-type jam developed using 50% of araticum pulp	The physical, chemical, and microbiological changes were within the standards established by legislation during one year of storage. Sensory aspects were not affected during the storage, reaching a score of 8 in the evaluated attributes (appearance, color, flavor, and aroma) by panelists during 0, 6, and 12 months of storage.	[108]
Jam	Araticum pulp (50 pulp/50 sugar ratio)	Araticum was a good fruit for jam development, allowing the consumption of the fruit throughout the year. Time, temperature, and packaging type influenced the quality of araticum jams during storage.	[90]
Jam	Extra-type jam with araticum pulp	Two jams were developed, conventional (sugar) and light (sucralose) with araticum pulp. Both showed physicochemical and microbiological stability during the six months of storage.	[109]
Juice	Araticum and cagaita juices were produced using 40% and 50% of pulp, respectively, and mixed juice was prepared with isolated pulp juices in different concentrations (16.33, 33, 50 and 67.33 %)	Juices produced with araticum and cagaita pulps (as well as their combinations) had higher antioxidant activity and phenolic compounds content and presented a greater acceptance by consumers than other formulations.	[95]
Juice	Araticum pulp juice (40% *v*/*v*) and mixed araticum (20% *v*/*v*) and cagaita (30% *v*/*v*) pulp juice	The juices developed showed considerable preservation of bioactive compounds. Araticum pulp juice showed higher total phenolic content and antioxidant activity than other formulations.	[94]
Juice	Araticum pulp (40%)	The araticum juice showed good sensory acceptance by consumers for all evaluated attributes (color, flavor, consistency, and general taste), presenting scores varying between hedonic terms “indifferent” and “moderately liked”.	[52]
Yogurt	Araticum pulp (5% *w*/*w*)	Greek yogurt with araticum pulp presented good acceptance levels, with average scores of 6.29 to 7.36, corresponding to the hedonic terms of “I liked slightly” and “I liked moderately”. 80 and 71% of consumers would buy araticum Greek yogurt after 7 and 28 days of storage at 4 °C, respectively.	[110]
Fermented dairy beverage	Araticum pulp (5.0, 7.5, 10.0, 12.5, 15.0, 17.5, and 20.0% *w*/*v*)	Formulations with higher concentrations of araticum pulp had higher percentages of ash, lipids, and proteins. Sensorially, all formulations were equally preferred by consumers, regardless of the percentage of araticum pulp.	[111]
Fermented milk drink	Araticum pulp (4, 8, 12, and 16%)	Regardless of the percentage of araticum pulp added, the fermented milk drinks showed the same acceptance regarding the evaluated parameters (color, aroma, flavor, acidity, viscosity, and appearance). The purchase intention was high for araticum fermented milk drink, with a percentage of 73% of purchase intention by the panelists.	[112]
Whey beverage	Araticum pulp freeze-dried (6% *w*/*w*)	The thermal treatments at different temperatures (70–100 °C) did not change the physicochemical properties (pH, total soluble solids, and ζ-potential), soluble sugar content, and FTIR spectra of the araticum whey beverage.	[113]
Snack bar	Araticum pulp flour (5, 10, 15, and 20 %) in replacement corn starch biscuit	The addition of araticum pulp flour to snack bars substantially improves the contents of dietary fiber, vitamin C, minerals, and antioxidant activity. The mean value score for the sensory attributes (appearance, aroma, flavor, texture, and overall acceptability) was 7, corresponding to the hedonic term “I liked moderately”.	[96]
Food bar	Araticum pulp flour (5, 10, 15, and 20%) in replacement of corn starch cake	The addition of araticum pulp flour promoted good acceptance of snack bars. Formulations with higher amounts (15 and 20%) of araticum pulp flour replacing corn starch obtained higher average scores concerning appearance, flavor, and texture attributes.	[114]
Food bar	Araticum pulp flour (20, 30, 40, and 50%) in replacement of oatmeal	Araticum pulp flour provided a product rich in dietary fiber, considerable amounts of vitamin C, carotenoids, minerals such as calcium and magnesium, and higher antioxidant activity. The addition of 50% araticum pulp flour improved the physical, chemical, and nutritional characteristics, in addition to favoring consumer sensory acceptance (hedonic values between 8—“I liked” and 9—“I liked very much”).	[97]
Milk caramel	Araticum pulp (16.6%)	The milk caramels with araticum pulp and the control milk caramels were equally accepted by the children, showing average acceptance scores between the hedonic terms “I liked moderately” and “I liked very much”, and acceptability indexes > 80%.	[115]

## 6. Conclusions

Araticum fruit has attracted increasing interest from researchers, consumers, and industrial sectors around the world due to its sensorial attractions, nutritional importance, and bioactive potential. This fruit has great importance for the population of the Cerrado region, since, in addition to being a source of various essential nutrients for the human diet, preparations with certain fruit parts have been used, since ancient times, to treat some pathological conditions. The data collected here has presented consistent scientific bases or even proven some of the applications of the araticum fruit in folk medicine. The edible part (pulp) and byproducts (peel and seeds) from araticum fruit contain several bioactive compounds, particularly phenolic compounds, alkaloids, annonaceous acetogenins, carotenoids, phytosterols, and tocols that display different biological activities. Recent studies compiled here found that bioactive-rich extracts, obtained from different parts of araticum fruit, can act as potent antioxidants, anti-inflammatories, antiproliferatives, hepatoprotectives, antinociceptives, enzyme inhibitors, antibacterials, etc. The presence of these bioactive compounds can explain its biological effects obtained from *in vitro* assays and animal trials, as well as its effectiveness in folk medicine, demonstrating its promising potential for drug development to treat/manage various pathological conditions, including inflammation, Alzheimer’s disease, cancer, diabetes mellitus, obesity, dyslipidemia, pain, cutaneous wounds, and illnesses associated with foodborne pathogen-contaminated food, among others. Moreover, araticum seed extracts displayed an interesting insecticide potential and can be exploited for the development of biodefensives to control crop pests and the insect vectors of pathogens. Although science has made relative progress in the identification and quantification of bioactive compounds from the araticum fruit, as well as the determination of their bioactivities, in the last decade, there are still many scientific gaps to be filled. Unfortunately, the data available in the literature are still scarce and insufficient to confirm the beneficial claims in human health since few *in vivo* studies have been conducted, and no clinical studies have been carried out to date. Therefore, clinical and interventional studies with humans must be carried out to confirm the biological effects described by *in vitro* and *in vivo* studies and to better understand the real benefits of consuming this fruit and/or its bioactive-rich extracts on human health and well-being. Furthermore, toxicological studies should be carefully conducted with the isolated compounds and extracts from the non-edible parts of the fruit to determine the toxic dose and, thus, ensure the safety and well-being of consumers.

Consumer behavior studies conducted with food products developed with araticum pulp have shown that breads, fruit preserves, sweet pastes, jams, juices, yogurts, fermented dairy beverages, whey beverages, food bars, and caramels have good sensory acceptance and purchase intention, demonstrating that araticum fruit is a promising fruit for producing conventional or innovative healthy products since the addition of araticum pulp improves their sensory (e.g., appearance, aroma, and flavor), nutritional (e.g., increases the content of dietary fiber, vitamins, and minerals), and functional (e.g., enhances the antioxidant activity and increases the content of phenolic compounds and carotenoids) characteristics. The high sensory acceptability of food products developed with the araticum fruit, as well as its high content of bioactive compounds and wide range of biological activities, clearly demonstrate the great potential for economic exploitation of this native fruit from the Brazilian Cerrado. However, to enable the economic exploitation of this fruit, an appropriate method of vegetative propagation is necessary to produce uniform and quality seedlings. The propagation of this native Brazilian fruit tree can be done through seeds; however, due to seed dormancy and the difficulty of obtaining uniform plants in a short time, asexual or clonal vegetative propagation (e.g., micropropagation, grafting, and cutting) emerges as an alternative to overcome this obstacle. Therefore, to make the araticum a sustainable cash crop, it is necessary to expand genetic and agronomic studies, as well as efficient vegetative propagation techniques to develop technical plantings that present early fruiting, increased productivity, fruiting at different times of the year, and adaptability to different areas and edaphoclimatic conditions around the world.

The discoveries made so far demonstrate that the araticum can be a valuable source of bioactive compounds for use in food, medicinal, cosmetic, and agricultural applications. However, some gaps, particularly those related to planting/propagation mechanisms and the confirmation of the safety and beneficial effects of its consumption through clinical/toxicological studies, still need to be further explored in future studies.

## Figures and Tables

**Table 1:** A summary of studies showing the bioactive compounds found in araticum fruit.

Bioactive Compounds	Fruit Part	Sample Form	Technique Used	Major Findings	Ref.
Phenolic compounds	Pulp	Hydroethanolic extract (40% ethanol)	HPLC-ESI-MS/MS	There were two phenolic compounds tentatively identified: ferulic acid and catechin.	[13]
	Pulp	Hydroethanolic extract (70% ethanol) and its ethyl acetate fraction	HPLC-ESI-MS/MS	There were 12 phenolic compounds tentatively identified: *p*-coumaric, gallic acid, ferulic acid, apigenin, epicatechin, 2′,5-dimethoxyflavone, 3′,7-dimethoxy-3-hydroxyflavone, kaempferol-3-*O*-glucoside, kaempferol-3-*O*-rutinoside, quercetin-3-*O*-glucoside, procyanidin B2, and rutin.	[14]
	Pulp	Hydromethanolic extract (70% methanol)	HPLC-DAD	There were 12 phenolic compounds identified and quantified: gallic acid (1.89 mg/100 g fw), catechin (16.79 mg/100 g fw), chlorogenic acid (0.55 mg/100 g fw), caffeic acid (0.03 mg/100 g fw), vanillin (0.46 mg/100 g fw), *p*-coumaric acid (0.02 mg/100 g fw), ferulic acid (0.03 mg/100 g fw), trans-cinnamic acid (0.20 mg/100 g fw), *m*-coumaric acid (0.21 mg/100 g fw), *o*-coumaric acid (0.01 mg/100 g fw), quercetin (0.51 mg/100 g fw), and rutin (0.40 mg/100 g fw).	[15]
	Peel	Hydroethanolic extract (50% ethanol)	HPLC-ESI-MS/MS	There were 14 phenolic compounds identified and quantified: epicatechin (54.46–136.47 µg/g dw), rutin (38.02–58.53 µg/g dw), chlorogenic acid (9.11–16.83 µg/g dw), catechin (7.38–15.23 µg/g dw), ferulic acid (5.18–10.96 µg/g dw), vicenin-2 (1.48–2.60 µg/g dw), vanillin (1.10–3.47 µg/g dw), naringenin (1.00–2.15 µg/g dw), protocatechuic acid (1.03–1.64 µg/g dw), caffeic acid (0.38–1.22 µg/g dw), luteolin (0.49–0.91 µg/g dw), *p*-coumaric acid (0.13–0.49 µg/g dw), 4-hydroxybenzoic acid (0.19–0.49 µg/g dw), and vitexin (0.08–0.18 µg/g dw). There were 112 phenolic compounds tentatively identified, particularly derivatives of syringic acid, ferulic acid, protocatechuic acid, hydroxybenzoic acid, caffeic acid, vanillic acid, chlorogenic acid, *p*-coumaric acid, (epi)catechin, kuwanon G, quercetin, kaempferol, apigenin, neocarthamin, isorhamnetin, luteolin, naringenin, vanillin, and lariciresinol.	[16]
	Peel	Ethyl acetate and *n*-butanol fractions from the ethanolic extract	HPLC-ESI-MS/MS	There were eight phenolic compounds tentatively identified in ethyl acetate fraction: caffeoyl-glucoside, (epi)catechin, procyanidins B2 and C1, feruloyl-galactoside, quercetin-3-glucoside, kaempferol-3-*O*-rutinoside, and kaempferol-7-*O*-glucoside. There were five phenolic compounds tentatively identified in *n*-butanol fraction: caffeoyl-glucoside, chlorogenic acid, procyanidins B2, feruloyl-galactoside, and (epi)catechin.	[17]
	Peel	Proanthocyanidins-rich fraction obtained from sequential purification of ethanolic extract	HPLC-ESI-MS/MS	Oligomers of B-type procyanidin were tentatively identified, including dimer, trimer, tetramer, and pentamer.	[18]
	Peel	Ethanolic extract and its ethyl acetate fraction	HPLC-ESI-MS/MS	There were 12 phenolic compounds tentatively identified in the ethanolic extract: chlorogenic acid, procyanidin B2, (epi)catechin, quercetin-glucosylpentoside, rutin, quercetin-glucoside, quercetin-glucuronide, quercetin-pentoside, quercetin-rhamnoside, kaempferol-rhamnoside, diosmetin-glucoside, and quercetin. There were 17 phenolic compounds tentatively identified in ethyl acetate fraction: caffeoyl-glucoside, chlorogenic acid, (epi)catechin, procyanidins B2 and C1, feruloyl-galactoside, quercetin, quercetin-glucoside, quercetin-pentoside, quercetin-rhamnoside, quercetin-glucuronide, quercetin-glucosylpentoside, rutin, kaempferol-glucoside, kaempferol-rutinoside, kaempferol-rhamnoside, and diosmetin-glucoside.	[19]
	Seeds	Hydromethanolic extract (70% methanol)	HPLC-DAD	There were 11 phenolic compounds identified and quantified: gallic acid (135.6 mg/kg dw), catechin (35.1 mg/kg dw), chlorogenic acid (14.7 mg/kg dw), caffeic acid (40.5 mg/kg dw), vanillin (3.1 mg/kg dw), *p*-coumaric acid (188.5 mg/kg dw), ferulic acid (63.9 mg/kg dw), trans-cinnamic acid (102.6 mg/kg dw), *o*-coumaric acid (3822.5 mg/kg dw), quercetin (83.5 mg/kg dw), and rutin (2209.4 mg/kg dw).	[20]
	Seeds	Methanolic extract	HPLC-DAD	There were five phenolic compounds identified and quantified: caffeic acid (302 µg/mL), sinapic acid (248 µg/mL), rutin (493 µg/mL), *p*-coumaric acid (106 µg/mL), and ferulic acid (176 µg/mL).	[21]
	Pulp and seeds	Ethanolic extract	PS-MS	There were three phenolic compounds tentatively identified in the pulp: epicatechin, sinapaldehyde glucoside, and eleutheroside B. The phenolic compound quercetin-*O*-pentosylhexoside was tentatively identified in the seed.	[22]
	Peel and seeds	Methanol-acetone-water (7:7:6, *v*/*v*/*v*) extract	HPLC-ESI-MS/MS	There were 12 phenolic compounds found in the peel: catechin (579.40 µg/g extract), epicatechin (6221.63 µg/g extract), rutin (133.31 µg/g extract), chlorogenic acid (305.15 µg/g extract), *p*-coumaric acid (14.97 µg/g extract), and traces of quercetin, naringenin, protocatechuic acid, 4-hydroxybenzoic acid, vanillic acid, caffeic acid, and ferulic acid. There were 12 phenolic compounds found in the seed: catechin (8.58 µg/g extract), epicatechin (12.10 µg/g extract), rutin (5.51 µg/g extract), quercetin (21.11 µg/g extract), naringenin (0.31 µg/g extract), protocatechuic acid (9.98 µg/g extract), 4-hydroxybenzoic acid (0.68 µg/g extract), vanillic acid (4.63 µg/g extract), chlorogenic acid (16.41 µg/g extract), caffeic acid (13.50 µg/g extract), *p*-coumaric acid (2.43 µg/g extract), and ferulic acid (2.51 µg/g extract).	[23]
	Pulp, peel, and seeds	Aqueous extract	PS-MS	There were 19 phenolic compounds found in the pulp: 4-hydroxybenzoic acid, gallic acid, sinapic acid, *p*-coumaric acid methyl ester, ferulic acid, coumaric acid hexose, feruloyl glycoside, apigenin, epicatechin, quercetin, naringenin, quercetin hexoside, lariciresinol-glucopyranoside, tangeretin, 5-caffeoylquinic acid, syringic acid glucopyranoside, quercetin-3-*O*-rhamnosylpentoside, procyanidin dimer type A (isomer 2), and procyanidin trimer type B. There were 17 phenolic compounds found in the peel: 4-hydroxybenzoic acid, gallic acid, sinapic acid, *p*-coumaric acid methyl ester, ferulic acid, coumaric acid hexose, feruloyl glycoside, apigenin, epicatechin, quercetin, tangeretin, 5-caffeoylquinic acid, syringic acid glucopyranoside, (neo)poncirin, hydroxytyrosol hexoside (isomer 2), procyanidin dimer type B (isomer 3), and procyanidin dimer type A (isomer 2). There were 21 phenolic compounds found in the seed: 4-hydroxybenzoic acid, gallic acid, sinapic acid, *p*-coumaric acid methyl ester, ferulic acid, coumaric acid hexose, feruloyl glycoside, apigenin, epicatechin, quercetin, tangeretin, protocatechuic acid, *p*-coumaric acid, caffeoyl glucose, luteolin-glucopyranoside, naringenin, quercetin hexoside, lariciresinol-glucopyranoside, (neo)poncirin, hydroxytyrosol hexoside (isomer 2), and procyanidin dimer type B (isomer 3).	[24]
	Pulp, peel, and seeds	Methanol-acetone-water (7:7:6, *v*/*v*/*v*) extract	HPLC-ESI-MS/MS	There were 10 phenolic compounds found in the pulp: catechin (768.42 µg/g dw), epicatechin (661.81 µg/g dw), rutin (9.31 µg/g dw), quercetin (7.80 µg/g dw), protocatechuic acid (97.92 µg/g dw), gentisic acid (14.00 µg/g dw), chlorogenic acid (43.45 µg/g dw), caffeic acid (124.31 µg/g dw), *p*-coumaric acid (11.86 µg/g dw), and ferulic acid (53.71 µg/g dw). There were 10 phenolic compounds found in the peel: catechin (3526.78 µg/g dw), epicatechin (1632.90 µg/g dw), rutin (8.30 µg/g dw), quercetin (21.83 µg/g dw), protocatechuic acid (317.90 µg/g dw), gentisic acid (8.80 µg/g dw), chlorogenic acid (13.24 µg/g dw), caffeic acid (93.45 µg/g dw), *p*-coumaric acid (46.90 µg/g dw), and ferulic acid (65.40 µg/g dw). There were 10 phenolic compounds found in the seed: catechin (33.60 µg/g dw), epicatechin (25.40 µg/g dw), rutin (1.81 µg/g dw), quercetin (1.35 µg/g dw), protocatechuic acid (22.44 µg/g dw), gentisic acid (3.28 µg/g dw), chlorogenic acid (11.00 µg/g dw), caffeic acid (45.36 µg/g dw), *p*-coumaric acid (3.40 µg/g dw), and ferulic acid (16.40 µg/g dw).	[25]
Alkaloids	Pulp	Hydroethanolic extract (70% ethanol) and its ethyl acetate fraction	HPLC-ESI-MS/MS	There were three alkaloids tentatively identified: xylopine, stephalagine, and romucosine.	[14]
	Peel	Ethanolic extract	HR-ESI-MS and NMR	There were three alkaloids isolated and purified from the ethanolic extract: stephalagine, liriodenine, and atherospermidine.	[26]
	Peel	Ethanolic extract	HR-ESI-MS and NMR	The aporphine alkaloid stephalagine was isolated and purified from the ethanolic extract.	[27,28,29]
	Peel	Dichloromethane fraction from the ethanolic extract	HPLC-ESI-MS/MS	There were nine alkaloids tentatively identified: isopiline, isoboldine, isocorydine, anonaine, xylopine, stephalagine, nuciferine, liriodenine, and atherospermidine.	[30]
	Pulp and peel	Ethanolic extract	PS-MS	There were eight alkaloids tentatively identified in the pulp: asimilobine, roemerine, nornuciferine, *N*-methylcoclaurine, guattescidine, actinodaphnine, isoboldine, and *N*-methyllaurotetanine. There were eight alkaloids tentatively identified in the peel: reticuline, roemerine, nornuciferine, *N*-methylcoclaurine, guattescidine, actinodaphnine, isoboldine, and *N*-methyllaurotetanine.	[22]
Annonaceous acetogenins	Pulp	Hydroethanolic extract (70% ethanol) and its ethyl acetate fraction	HPLC-ESI-MS/MS	The annonaceous acetogenin annonacin was tentatively identified.	[14]
	Peel	Hydroethanolic extract (50% ethanol)	HPLC-ESI-MS/MS	There were 21 annonaceous acetogenins tentatively identified, including isomers of annohexocin or murihexocin, annonisin, squamostatin A, annomuricin A, annoglaucin, 9-oxo-asimicinone, squamostatin E or squamocin F, montanacin A, mosinone A, squamoxinone or annomutacin, and annoreticuin.	[16]
	Peel	Dichloromethane fraction from the ethanolic extract	HPLC-ESI-MS/MS	There were four annonaceous acetogenins tentatively identified: bullatanocin, squamocin, annomontacin, and desacetyluvaricin/isodesacetyluvaricin.	[30]
	Pulp and seeds	Methanolic extract	LC-HRMS	There were two annonaceous acetogenins found in the pulp: annonacin (0.33 mg/g dw) and squamocin (0.40 mg/g dw). There were two annonaceous acetogenins found in the seed: annonacin (5.90 mg/g dw) and squamocin (142 mg/g dw).	[31]
	Pulp, peel, and seeds	Ethanolic extract	PS-MS	There were 17 annonaceous acetogenins tentatively identified in the pulp: squamostolide, corepoxylone, montalicin A, annonacinone, annodienin, montanacin E, squamolinone, annomontacin, itrabin, montanacin-J + 34-epi, bullatacinone, 9-OH-asimicinone, otivarin, parisin, 24-acetylguanacone, guanaconetin-4, and robustocin. There were nine annonaceous acetogenins tentatively identified in the peel: artemoin-A + B + C + D, dieporeticanin-2, annocatacin B, montalicin A, annonacinone, annodienin, montanacin E, squamolinone, and bullatencin. There were 22 annonaceous acetogenins tentatively identified in the seeds: montalicin A, annonacin, squamocin B, desacetyluvaricin, squamocin, muricatatin C, guanacone, tucumanin, montanacin H + 34-epi, murihexocin C, 9-oxo-asimicinone, coriheptocin B, annomontacin, itrabin, montanacin-J + 34-epi, bullatacinone, 9-OH-asimicinone, otivarin, parisin, 24-acetylguanacone, guanaconetin-4, and salzmanolind.	[22]
Carotenoids	Pulp	Acetone extract	HPLC-DAD	There were three carotenoids identified and quantified: lycopene (0.02 mg/100 g fw), α-carotene (2.98 mg/100 g fw), and β-carotene (1.97 mg/100 g fw).	[10]
	Pulp	Acetone extract	HPLC-DAD	There were two carotenoids identified and quantified: all-trans-α-carotene (1.55–1.98 mg/100 g fw) and all-trans-β-carotene (0.86–1.58 mg/100 g fw).	[32]
Phytosterols	Seeds	Chloroform-methanol-water (2:1:0.8, *v*/*v*/*v*) extract	GC-FID	There were three phytosterols identified and quantified: campesterol (204.32 mg/kg dw), stigmasterol (179.25 mg/kg dw), and β-sitosterol (300.02 mg/kg dw).	[33]
Tocols	Pulp	Fruit pulp was extracted with heated ultrapure water-isopropanol-hexane containing 0.05% of BHT-solvent mixture (hexane-ethyl acetate, 85:15, *v*/*v*) (4:10:1:25, *v*/*v*/*v*/*v*)	HPLC-FLD	There were two tocols identified and quantified: α-tocotrienol (332.94 µg/100 g fw) and α- tocopherol (163.11 µg/100 g fw).	[10]
	Seeds	Chloroform-methanol-water (2:1:0.8, *v*/*v*/*v*) extract	HPLC-FLD	There were four tocols identified and quantified: α-tocopherol (12.02 mg/kg dw), β- tocopherol (3.30 mg/kg dw), γ-tocopherol (123.42 mg/kg dw), and δ-tocopherol (0.16 mg/kg dw).	[33]

BHT: Butylhydroxytoluene, dw: dry weight, fw: fresh weight, GC-FID: Gas Chromatography with Flame Ionization Detection, HPLC-DAD: High-Performance Liquid Chromatography coupled with Diode Array Detector, HPLC-ESI-MS/MS: High-Performance Liquid Chromatography coupled with a Mass Spectrometry with an Electrospray Ionization source, HPLC-FLD: High-Performance Liquid Chromatography coupled to Fluorescence Detector, HR-ESI-MS: High-Resolution Mass Spectrometry with Electrospray Ionization, LC-HRMS: Liquid Chromatography with High-Resolution Mass Spectrometry, NMR: Nuclear Magnetic Resonance, and PS-MS: Paper Spray Ionization Mass Spectrometry.

**Table 2:** A summary of studies showing the biological activities of extracts from edible parts and by-products of araticum fruit.

Bioactivity	Fruit Part	Sample Form	Method/Model	Major Findings	Related Compounds	Ref.
Antioxidant	Pulp	Hydroethanolic extract (46% ethanol)	DPPH, TEAC, and ORAC based *in vitro* assays	Fruit pulp showed 609.58, 683.65, and 1593.72 µmol TE/g dw for DPPH, TEAC, and ORAC assays, respectively.	Phenolic compounds (compounds were not identified)	[36]
	Pulp	Hydroethanolic extract (40% ethanol)	DPPH and TEAC based *in vitro* assays	Fruit pulp showed 150.13 and 184.81 µmol TE/g dw for DPPH and TEAC assays, respectively.	Phenolic compounds (see Table 1)	[13]
	Pulp	Methanolic (for DPPH) and aqueous (for TEAC and ORAC) extracts	DPPH, TEAC, and ORAC based *in vitro* assays	Fruit pulp showed 306.04, 231.79, and 902.27 µmol TE/g dw for DPPH, TEAC, and ORAC assays, respectively.	Phenolic compounds (compounds were not identified)	[51]
	Pulp	Hydroethanolic extract (70% ethanol) and its ethyl acetate fraction	DPPH and TEAC based *in vitro* assays	Ethyl acetate fraction (IC_50_ 57.80 µg/mL and 192.61 µmol TE/g dw for DPPH and TEAC) showed higher antioxidant activity than hydroethanolic extract (IC_50_ 182.54 µg/mL and 94.66 µmol TE/g dw for DPPH and TEAC).	Phenolic compounds (see Table 1)	[14]
	Pulp	Combination of hydromethanolic (50% methanol) and hydroacetonic (70% acetone) extracts	DPPH, TEAC, and β-carotene/linoleic acid based *in vitro* assays	Fruit pulp showed IC_50_ 148.59 g pulp fw/g DPPH, 132.16 µmol TE/g fw, and 51.28% protection for DPPH, TEAC, and β-carotene/linoleic acid assays, respectively.	Phenolic compounds (compounds were not identified)	[52]
	Pulp	Combination of hydromethanolic (50% methanol) and hydroacetonic (70% acetone) extracts	DPPH, TEAC, β-carotene/linoleic acid, TBARS, phosphomolybdenum complex, and reducing power based *in vitro* assays	Fruit pulp showed 19.51, 10.65, 2.91, 0.04, 39.04, and 17.77 mg BHT equivalents/g fw by DPPH, TEAC, β-carotene/linoleic acid, TBARS, reducing power, and phosphomolybdenum complex assays, respectively.	Phenolic compounds (see Table 1)	[15]
	Peel	Hydroethanolic extract (50% ethanol)	DPPH, TEAC, and ORAC based *in vitro* assays	Under optimal extraction conditions, fruit peel showed 514.57, 613.76, and 525.41 µmol TE/g dw for DPPH, TEAC, and ORAC assays, respectively.	Phenolic compounds (see Table 1)	[16]
	Peel	Ethanolic extract and its fractions (hexane, dichloromethane, ethyl acetate, *n*-butanol, and aqueous)	DPPH, FRAP, and ORAC based *in vitro* assays	Ethyl acetate and *n*-butanol fractions showed the highest antioxidant activities (DPPH IC_50_ 1.5 and 0.8 μg/mL, ORAC 3355 and 2714 μmol TE/g, and FRAP 888 and 921μmol TE/g, respectively).	Phenolic compounds (see Table 1)	[17]
	Peel	Procyanidin B-rich fraction and ethyl acetate fraction from ethanolic extract	DPPH, FRAP, and ORAC based *in vitro* assays, opsonized zymosan-induced macrophages, and Fe^2+^-ascorbate-induced lipid peroxidation in rats’ liver	Procyanidin B-rich fraction showed the highest antioxidant activities (DPPH IC_50_ 5.4 μg/mL, ORAC 9691 μmol TE/g, and FRAP 2438 μmol TE/g). ↓ ROS production in zymosan-induced macrophages (0.1–10 and 1–10 μg/mL for procyanidin and ethyl acetate fractions, respectively). ↓ Liver lipid peroxidation (0.5–50 μg/mL). ↑ Total antioxidant capacity in the liver (5–50 μg/mL).	Procyanidins B	[18]
	Seeds	Combination of hydromethanolic (50% methanol) and hydroacetonic (70% acetone) extracts	TEAC, FRAP, and β-carotene/linoleic acid based *in vitro* assays	Fruit seeds showed 205 µmol TE/g dw, 659 µmol FeSO_4_/g dw, and 90.7% protection for TEAC, FRAP, and β-carotene/linoleic acid assays, respectively.	Compounds were not identified	[53]
	Seeds	Oil	DPPH-based *in vitro* assay	Seed oil showed IC_50_ of 8.22 g oil/g DPPH.	Particularly phytosterols (campesterol, stigmasterol, and β-sitosterol)	[33]
	Peel and seeds	Methanol-acetone-water (7:7:6, *v*/*v*/*v*) extract	DPPH, TEAC, and ORAC based *in vitro* assays	Fruit peel extract showed 1065.00, 2022.13, and 4643.78 µmol TE/g dw for DPPH, TEAC, and ORAC assays, respectively. Fruit seeds extract showed 917.00, 190.54, and 5166.30 µmol TE/g dw for DPPH, TEAC, and ORAC assays, respectively.	Phenolic compounds (see Table 1)	[23]
	Pulp, peel, and seeds	Methanol-acetone-water (7:7:6, *v*/*v*/*v*) extract	DPPH, TEAC, and ORAC based *in vitro* assays	Fruit pulp showed 132.73, 214.38, and 259.26 µmol TE/g dw for DPPH, TEAC, and ORAC assays, respectively. Fruit peel showed 189.32, 292.28, and 448.29 µmol TE/g dw for DPPH, TEAC, and ORAC assays, respectively. Fruit seeds showed 71.66, 94.81, and 260.22 µmol TE/g dw for DPPH, TEAC, and ORAC assays, respectively.	Phenolic compounds (see Table 1)	[25]
	Pulp, peel, and seeds	Combination of hydromethanolic (50% methanol) and hydroacetonic (70% acetone) extracts	DPPH, TEAC, and FRAP based *in vitro* assays	Fruit pulp showed 3025.67–5222.50 µmol DPPH/g fw, 23.16–93.76 µmol TE/g fw, and 39.08–99.34 µmol FeSO_4_/g fw for DPPH, TEAC, and FRAP assays, respectively. Fruit peel showed 631.77–3373.34 µmol DPPH/g fw, 95.52–367.63 µmol TE/g fw, and 7.18–273.95 µmol FeSO_4_/g fw for DPPH, TEAC, and FRAP assays, respectively. Fruit seeds showed 4038.71–24,627.17 µmol DPPH/g fw, 18.69–93.74 µmol TE/g fw, and 9.21–48.57 µmol FeSO_4_/g fw for DPPH, TEAC, and FRAP assays, respectively.	Phenolic compounds (see Table 1)	[24]
Anti-Alzheimer	Pulp	Aqueous extract	Juglone-induced oxidative stress in wild-type (N2) strains of *Caenorhabditis elegans* and CL2006 strains of *C. elegans* expressing the Aβ1–42 peptide in muscle tissue	↑ Survival rates of worms (N2 strains) exposed to juglone (32.2% survival at 1 mg/mL). ↓ Worms (CL2006 strains) paralyzed (decrease in 13.4% paralysis at 1 mg/mL).	Compounds were not identified	[54]
	Peel	Ethanolic extract, alkaloidal fraction, and isolated alkaloids	*In vitro* cholinesterase activity assay	Ethanolic extract and alkaloidal fraction displayed a low inhibition profile against AChE and BChE. Liriodenine showed the highest AChE inhibitory activity followed by atherospermidine and stephalagine (IC_50_ 2.7, 5.6, and 12.6 µg/mL, respectively). Stephalagine showed de higher BChE inhibitory activity followed by liriodenine and atherospermidine (IC_50_ 32.7, 46.2, and 61.4 µg/mL, respectively).	Aporphine alkaloids, particularly stephalagine, liriodenine, and atherospermidine	[26]
	Seeds	Methanolic extract	*In vitro* cholinesterase activity assay	Extract inhibited 45% of AChE activity at 1.5 mg/mL.	Phenolic compounds, particularly rutin, caffeic, sinapic, *p*-coumaric, and ferulic acids	[21]
Anticancer	Pulp	Hydroethanolic extract (70% ethanol)	*In vitro* antiproliferative activity against 6 human cancer cell lines: UA251, MCF-7, PC-3, OVCAR-3, HT-29, and HEP-G2	Extract showed a significant antiproliferative effect against only the glioblastoma line (UA251) with IC_50_ of 21.34 µg/mL. Extract was highly selective for the cancer cell line (IC_50_ > 100 µg/mL for non-tumor cell line HaCaT).	Annonaceous acetogenins, phenolic compounds, and alkaloids (see Table 1)	[14]
	Peel	Alkaloid and acetogenin-rich fraction from ethanolic extract	*In vitro* antiproliferative activity against HEP-G2 cells	↓ HEPG2 cells viability, proliferation, and migration at 50 µg/mL. ↓ PCNA and EGFR expression in the HEPG2 cells at 50 µg/mL. ↑ Intracellular Ca^2+^ in the HEPG2 cells at 50 µg/mL.	Alkaloids and annonaceous acetogenins (see Table 1)	[30]
	Seeds	Methanolic extract	*In vitro* antiproliferative activity against 10 human cancer cell lines: UACC-62, UA251, MCF-7, NCI-H460, 786–0, PC-3, NCI-ADR/RES, OVCAR-3, HT-29, and K562	Seeds extract was highly active against all human cancer cells (IC_50_ 0.01–8.90 µg/mL). HT-29, NCI-H460, UA251, and NCI-ADR/RES were the cancer cells more sensitive to the extract (IC_50_ 0.01, 0.04, 0.06, and 0.25 µg/mL, respectively).	Phenolic compounds, particularly rutin, caffeic, sinapic, *p*-coumaric, and ferulic acids	[21]
	Peel and seeds	Methanol-acetone-water (7:7:6, *v*/*v*/*v*) extract	*In vitro* antiproliferative activity against 8 human cancer cell lines: UA251, MCF-7, NCI-H460, PC-3, NCI-ADR/RES, OVCAR-3, HT-29, and K562	Seeds extract (TGI 5.36–76.77 µg/mL) showed the best cytostatic effect against all cancer cells compared to peel extract (TGI 37.64–230.12 µg/mL). Seeds extract was highly active against NCI-ADR/RES, PC-3, and OVCAR-3 (TGI 5.36, 16.60, and 20.93 µg/mL, respectively). Both extracts were not capable to inhibit NCI-H460.	Phenolic compounds (see Table 1)	[23]
Antidiabetic	Peel	Ethanolic extract and its fractions (hexane, dichloromethane, ethyl acetate, *n*-butanol, and aqueous)	*In vitro* inhibitory activities against α-amylase, α-glucosidase, and non-enzymatic glycation	Ethyl acetate and *n*-butanol fractions showed the highest inhibitory activities against α-amylase (IC_50_ 4.5 and 1.7 μg/mL), α-glucosidase (IC_50_ 554.5 and 787.8 μg/mL), and glycation (IC_50_ 14.3 and 16.0 μg/mL).	Phenolic compounds (see Table 1)	[17]
	Peel	Procyanidin B-rich fraction and ethyl acetate fraction from ethanolic extract	Antiglycation based *in vitro* assays	↓ AGEs production in the BSA-fructose, BSA-methylglyoxal, and arginine-methylglyoxal models. ↓ AGEs-induced protein crosslinks, protein-bound carbonyls group formation, and protein thiol group oxidation. Attenuated the decrease in glycated CAT activity.	Procyanidins B	[18]
Anti-obesity	Peel	Ethanolic extract, dichloromethane fraction, and isolated stephalagine	*In vitro* inhibitory activity against pancreatic lipase and cytotoxicity with Vero cells	Stephalagine showed the highest inhibitory activity against pancreatic lipase (IC_50_ 8.35 μg/mL), followed by ethanolic extract and dichloromethane fraction (IC_50_ 104.50 and 108.10 μg/mL, respectively). Stephalagine and ethanolic extract showed low cytotoxicity (CC_50_ 353 and 331 μg/mL, respectively).	Stephalagine	[29]
Antidyslipidemic and hepatoprotective	Peel	Ethanolic extract (EE) and its ethyl acetate fraction (EAF)	Triton WR-1339-induced hyperlipidemic C57BL/6 mice pretreated for 12 days with 10–100 mg EE or EAF/kg bw	↑ Plasma HDL-C (EE and EAF at 30 mg/kg). ↓ Hepatic triglycerides and total cholesterol (EE at 10–100 mg/kg). ↓ Hepatic lipid peroxidation (all EAF doses and EE at 100 mg/kg). ↓ Hepatic protein carbonylation (EAF at 10 mg/kg). Maintained the hepatic total thiol content (EAF at 30 mg/kg). ↑ Hepatic SOD and CAT activities (EE at 10 mg/kg). Restored hepatic GPx and GR activities and GSH levels (100 mg/kg). ↑ Hepatic G6PD activity (all EE doses and EAF at 30 and 100 mg/kg).	Phenolic compounds (see Table 1)	[19]
Hepatoprotective and antioxidant	Peel	*n*-Butanol fraction from ethanolic extract	Streptozotocin-induced diabetic Wistar rats receiving 25, 50, or 100 mg *n*-butanol fraction/kg bw for 30 days	↓ Serum ALT and AST activities, hepatic lipid peroxidation, and hepatic SOD and CAT contents (all treatments). ↓ Glycemia and ↑ hepatic GR activity (only 100 mg/kg/day). ↓ Serum ALP activity and ↑ hepatic total antioxidant activity (only 50 mg/kg/day). ↓ Hepatic protein carbonylation, GPx, and nitrotyrosine contents, and ↑ hepatic GSH content (only 50 and 100 mg/kg/day). ↓ Hepatic iNOS content (only 25 and 50 mg/kg/day).	Phenolic compounds, particularly chlorogenic acid, (epi)catechin, procyanidin B2, feruloyl-galactoside, and caffeoyl-glucoside	[55]
Antinociceptive and anti-inflammatory	Peel	Stephalagine isolated from ethanolic extract	C57BL/6/J mice receiving 0.1–1.0 mg/kg bw	↓ Formalin-induced nociception at 0.3–1.0 mg/kg bw (↓ paw licking time). ↓ Cinnamaldehyde-induced nociception when administered by oral route at 1.0 mg/kg bw (↓ paw licking time). ↓ Capsaicin-induced nociception (↓ paw licking time) and inflammation (↓ paw edema) when administered by oral route at 1.0 mg/kg bw. ↓ Capsaicin- and cinnamaldehyde-mediated Ca^2+^ influx (↓ activation of TRPV1 and TRPA1 channels). No alteration to the animals’ locomotor activity at 1.0 mg/kg bw.	Stephalagine	[28]
	Peel	Stephalagine isolated from ethanolic extract	Monosodium urate crystals-induced gout C57BL/6/J mice receiving 1 mg/kg bw	↓ Mechanical allodynia, spontaneous nociception, and cold hypersensitivity. ↓ Articular edema, MPO activity, IL-1β level, and neutrophil infiltration. Absence of hepatic (maintained the serum ALT and AST activities) or kidney (maintained the serum urea and creatinine levels) damage. No signals of toxicity (absence of abnormalities in the diet, sudden changes in body weight, changes in the hair, feces, behavior, and macroscopic anatomy of the mice).	Stephalagine	[27]
	Peel	Ethyl acetate fraction from ethanolic extract	LPS-induced macrophages and C57BL/6/J mice receiving 30 mg ethyl acetate fraction/kg bw	↓ IL-6 and NO in the LPS-induced macrophages. ↓ Glutamate-induced nociception (↓ paw licking time). Reverted the early and late hyperalgesia induced by CFA. ↓ CFA-induced cold nociceptive responses and paw edema in the acute phase. ↓ MPO activity and inflammatory cell infiltration in the paw tissue. No alteration to the animals’ locomotor activity.	Phenolic compounds, particularly caffeoyl-glucoside, (epi)catechin, procyanidins B2 and C1, feruloyl-galactoside, quercetin-3-glucoside, kaempferol-3-*O*-rutinoside, and kaempferol-7-*O*-glucoside	[56]
Anti-inflammatory	Pulp	Whole pulp	Wistar rats receiving 3.214 mL pulp/kg bw for 30 days	↓ Total leukocytes.	Compounds were not identified	[57]
Healing of cutaneous wounds	Peel	Combination of ethyl acetate and *n*-butanol fractions (1:1) from ethanolic extract (Polyphenol-rich fraction (PEF))	C57BL/6 mice topically treated with an ointment containing 2–6% PEF for 7 days	↑ Wound closure and deposition of types I and III collagen fibers. ↓ Neutrophils and macrophage activation (↓ MPO and NAG activities). ↑ Hemoglobin level and blood vessels on day 4 at 6% PEF.	Phenolic compounds (see Table 1)	[58]
	Peel	Combination of ethyl acetate and *n*-butanol fractions (1:1) from ethanolic extract (Polyphenol-rich fraction (PEF))	BALB/c mice topically treated with an ointment containing 4% PEF for 21 days	Accelerated wound closure. ↓ Neutrophils activation (↓ MPO and NAG activities) and lipid peroxidation. ↑ Number of mast cells and deposition of types I and III collagen fibers in wounds. ↑ MMP-2 and MMP-9 activities. Improved the antioxidant defense and dermis and epidermis organization during healing.	Phenolic compounds (see Table 1)	[59]
	Peel	Combination of ethyl acetate and *n*-butanol fractions (1:1) from ethanolic extract (Polyphenol-rich fraction (PEF))	BALB/c mice topically treated with an ointment containing 4% PEF for 7 days	Accelerated wound closure. ↓ Neutrophils activation (↓ MPO activity). ↑ Deposition of types I and III collagen fibers in wounds.	Phenolic compounds (see Table 1)	[60]
	Peel and seeds	Methanol-acetone-water (7:7:6, *v*/*v*/*v*) extract	Scratch assay with HaCaT cells	Seeds extract (3.6 µg/mL) promoted cell migration, leading to slot closure (73%). Peel extract (18–36 µg/mL) strongly inhibited cell migration (0.6–3.8% slot closure).	Phenolic compounds (see Table 1) and other compounds unidentified	[23]
Antibacterial	Pulp	Hydroethanolic extract (40% ethanol)	*In vitro* antibacterial activity against 4 potentially pathogenic bacteria *Staphylococcus aureus* (ATCC 25923), *Bacillus cereus* (ATCC 11778), *Escherichia coli* (ATCC 25922), and *Salmonella enteritidis* (ATCC 13076)	Extract was more active against gram-positive bacteria (MIC 12.5 and 25.0 mg/mL for *B. cereus* and *S. aureus*, respectively).	Phenolic compounds (see Table 1)	[13]
	Pulp, peel, and seeds	Hydroethanolic extract (70% ethanol)	*In vitro* antibacterial activity against Oxacillin Resistant *Staphylococcus aureus* (ORSA) and *S. aureus* (ATCC 6538)	Peel and pulp extracts were highly active against *S. aureus* (ATCC 6538) (MIC 6.25 and 12.5 mg/mL, respectively). All fruit part extracts were active against ORSA (MIC 25–50 mg/mL).	Alkaloids, flavonoids, tannins, and saponins (compounds were not identified)	[61]
Insecticide	Seeds	Hexane, chloroform, methanolic (defatted with hexane or dichloromethane) extracts and hexane, hydromethanolic, ethyl acetate, and chloroform fractions from methanolic extract defatted with dichloromethane	*Aedes aegypti* larvae	Hexane, dichloromethane, and methanolic (defatted with hexane) extracts and hexane fraction at 1 mg/mL controlled third-instar larvae (up to 100% mortality). Methanolic extract (defatted with hexane) was the most active followed by dichloromethane extract, hexane fraction, and hexane extract (IC_50_ 0.100, 0.185, 0.433, and 0.507 mg/mL, respectively).	Mainly annonaceous acetogenins (compounds were not identified)	[62]
	Seeds	Chloroform-methanol (2:1) extract	Rice stalk stink bug nymphs (*Tibraca limbativentris*)	Topical application (0.5–8% extract) reduced mobility and controlled second-instar nymphs (up to 81% mortality).	Mainly annonaceous acetogenins (compounds were not identified)	[63]
	Seeds	Chloroform-methanol (2:1) extract	Soybean looper eggs and caterpillars (*Chrysodeixis includens*)	Topical application (0.5–8% extract) controlled first-, third-, and fifth-instar caterpillars (up to 93.3% mortality). ↑ Mortality rates for first- and third-instar caterpillars fed treated leaves, reducing the number of caterpillars that completed their development.	Mainly annonaceous acetogenins (compounds were not identified)	[64]
	Seeds	Methanolic extract	Brown stink bug nymphs (*Euschistus heros*)	Control of third-instar nymphs at 0.5–4.0% extract.	Compounds were not identified	[65]
	Seeds	Methanolic extract	Brown stink bug adults (*Euschistus heros*)	Population control of *E. heros* in soybean crop after 7 days of application of extract at 2%.	Compounds were not identified	[66]

↑: increase, ↓: reduction, 786–0: kidney cancer cell line, AChE: acetylcholinesterase, AGEs: advanced glycation end products, ALP: alkaline phosphatase, ALT: alanine aminotransferase, AST: aspartate aminotransferase, BChE: butyrylcholinesterase, BHT: butylhydroxytoluene, BSA: bovine serum albumin, bw: body weight, CAT: catalase, CFA: complete Freund’s adjuvante, DPPH: DPPH radical scavenging activity, dw: dry weight, EGFR: epidermal growth factor receptor, FRAP: ferric reducing antioxidant power, fw: fresh weight, G6PD: glucose 6-phosphate dehydrogenase, GPx: glutathione peroxidase, GR: glutathione reductase, GSH: glutathione, HaCaT: spontaneously transformed keratinocytes, HDL-C: high-density lipoprotein cholesterol, HEP-G2: liver cancer cell line, HT-29: colorectal cancer cell line, IC_50_: extract concentration that resulted in a 50% reduction in the enzymatic activity/cell proliferation/radical concentration to the untreated control, IL: interleukins, iNOS: inducible nitric oxide synthase, K562: leukemia cell line, LPS: lipopolysaccharide, MCF-7: breast cancer cell line, MIC: minimum inhibitory concentration, MMP: metalloproteinases, MPO: myeloperoxidase, NAG: N-acetyl-β-D-glycosaminidase, NCI-ADR/RES: ovarian cancer cell line (expressing the phenotype of multiple drug resistance), NCI-H460: lung cancer cell line, NO: nitric oxide, ORAC: oxygen radical absorbance capacity, OVCAR-3: ovarian cancer cell line, PC-3: prostate cancer cell line, PCNA: proliferating cell nuclear antigen, ROS: reactive oxygen species, SOD: superoxide dismutase, TBARS: thiobarbituric acid reactive substances, TE: Trolox equivalents, TEAC: Trolox equivalent antioxidant capacity, TGI: concentration required to completely inhibit cell growth, TRPA1: transient receptor potential ankyrin 1 channel, TRPV1: transient receptor potential vanilloid 1 channel, UA251: glioblastoma cell line, UACC-62: melanoma cell line.

## Data Availability

Not applicable.
